# Supplementary material for: Syntaxin-6, a Reliable Biomarker for Predicting the Prognosis of Patients with Cancer and the Effectiveness of Immunotherapy
Source: Cancers (Basel). 2022 Dec 21;15(1):27. doi: 10.3390/cancers15010027 (PMC9817965; doi:10.3390/cancers15010027)
Supplement: Supplementary file 1 [file cancers-15-00027-s001.zip › cancers-2083385-File S1.pdf]

Supplementary File S1

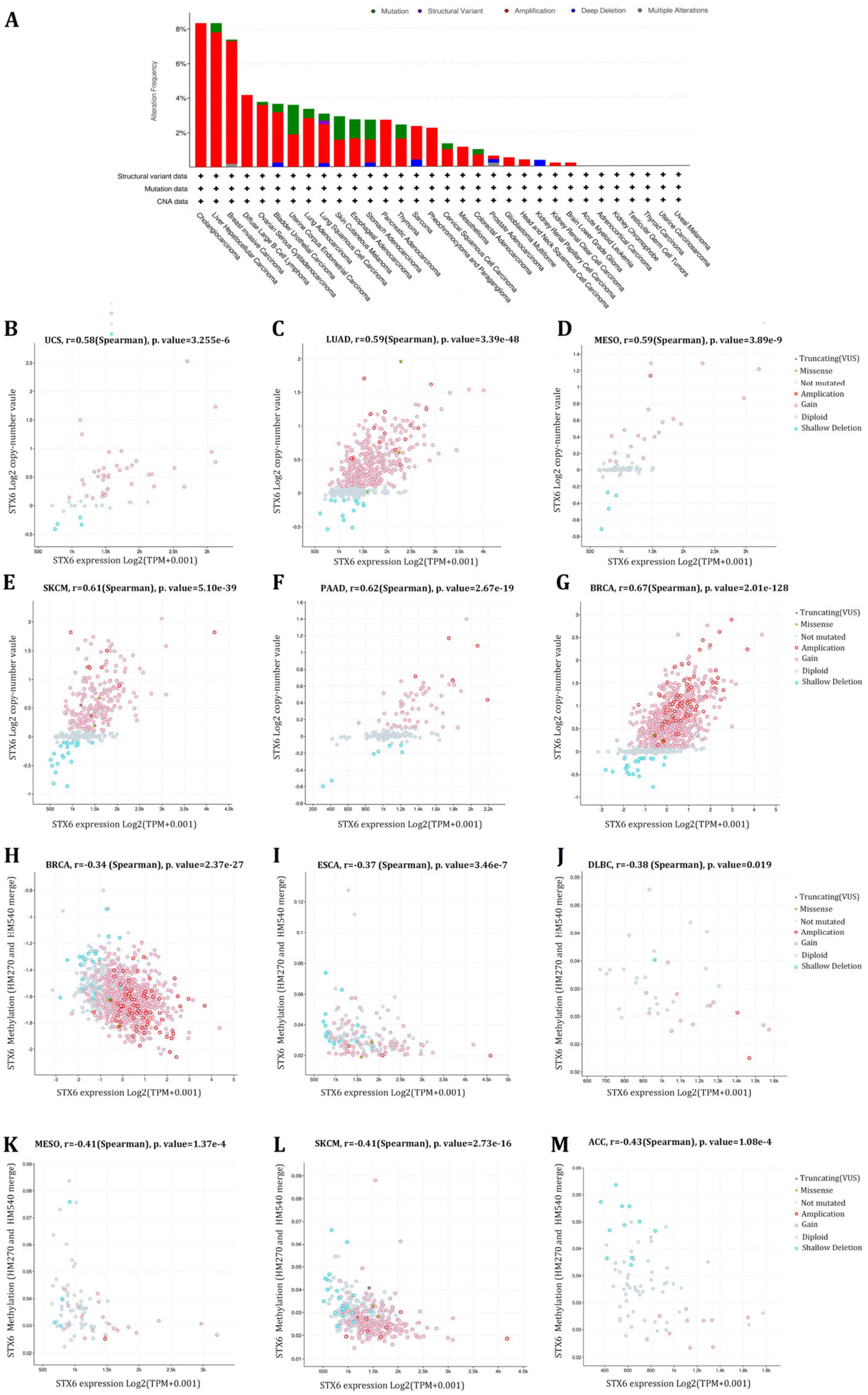

Figure S1. STX6 has a high mutation rate in tumor genome, especially in cholangiocarcinoma, LIHC or breast invasive cancer, and amplication is the main mutation (**A**). The expression level of STX6 was positive correlations with copy number variation (CNV): UCS, LUAD , MESO, SKCM, PAAD, and BRCA (**B-G**), whereas inversely linked with the amount of promoter methylation in BRCA, ESCA, DLBC, MESO, SKCM, and ACC (**H-M**).

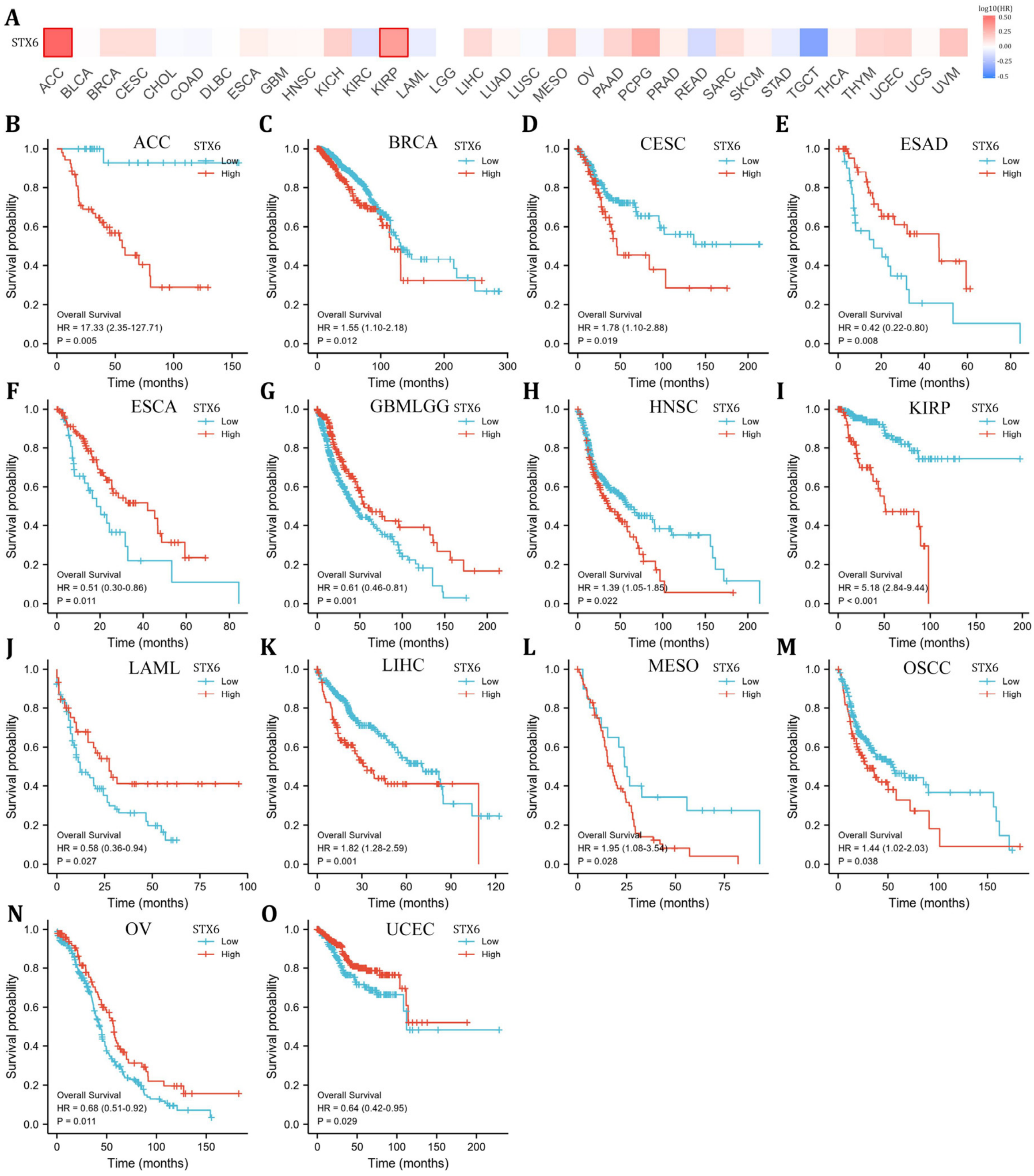

Figure S2: STX6 was a poor predictor of patient survival in ACC, BRCA, CESC, HNSC, KIRP, LIHC, MESO, and OSCC among these malignancies (**B, C, D, H, I, K, L, M**). STX6 enhanced the results of individuals with ESAD, ESCA, GBMLGG, LAML, OV, and UCEC (**E, F, G, J, N, O**), meanwhile (TCGA database).

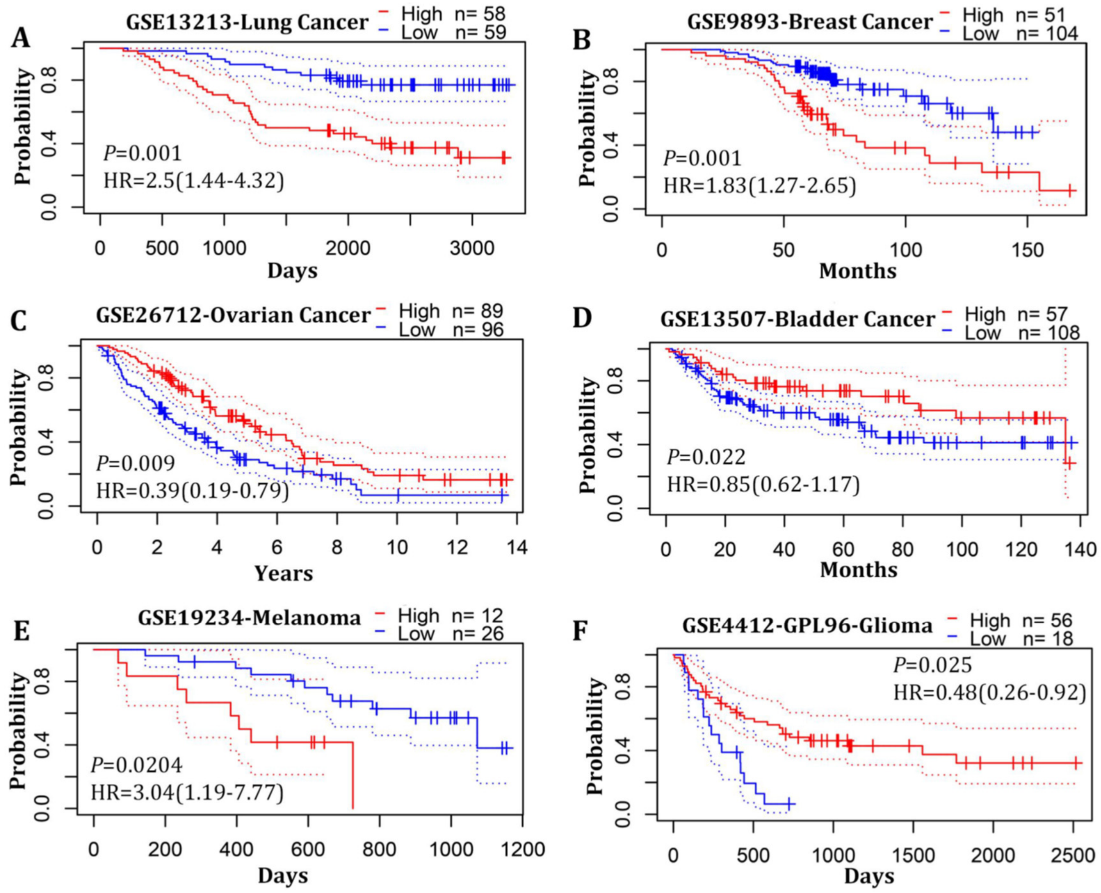

Figure S3: STX6 was a poor predictor of patient survival in lung cancer (**A**), Breast Cancer (**B**) and Melanoma (**E**), while was a favor predictor for patients with Ovarian Cancer (**C**), Bladder Cancer (**D**) and Glioma (**F**) (GEO database).

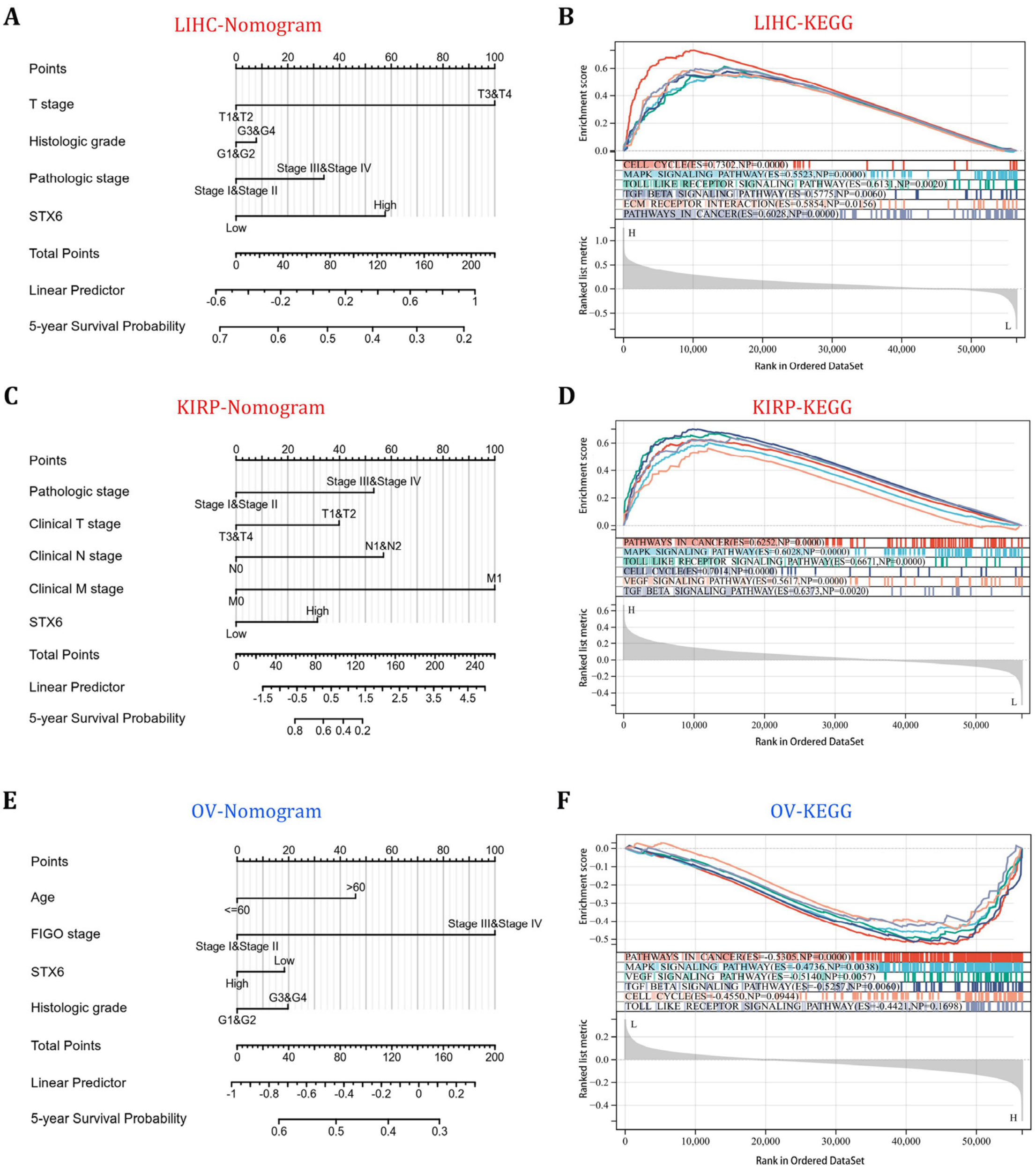

Figure S4: STX6 can be used as a powerful predictor of 5-year survival rate in patients with LIHC (**A**), KIRP (**C**) or OV (**E**). KEGG pathway enrichment analysis indicated that STX6 was significantly associated with pathways in cancer, cell cycle, EMT, VEGF signal pathway, TGF BEBTA pathway and Toll-like receptor pathways in LIHC (**B**), KIRP (**D**) and OV (**F**).

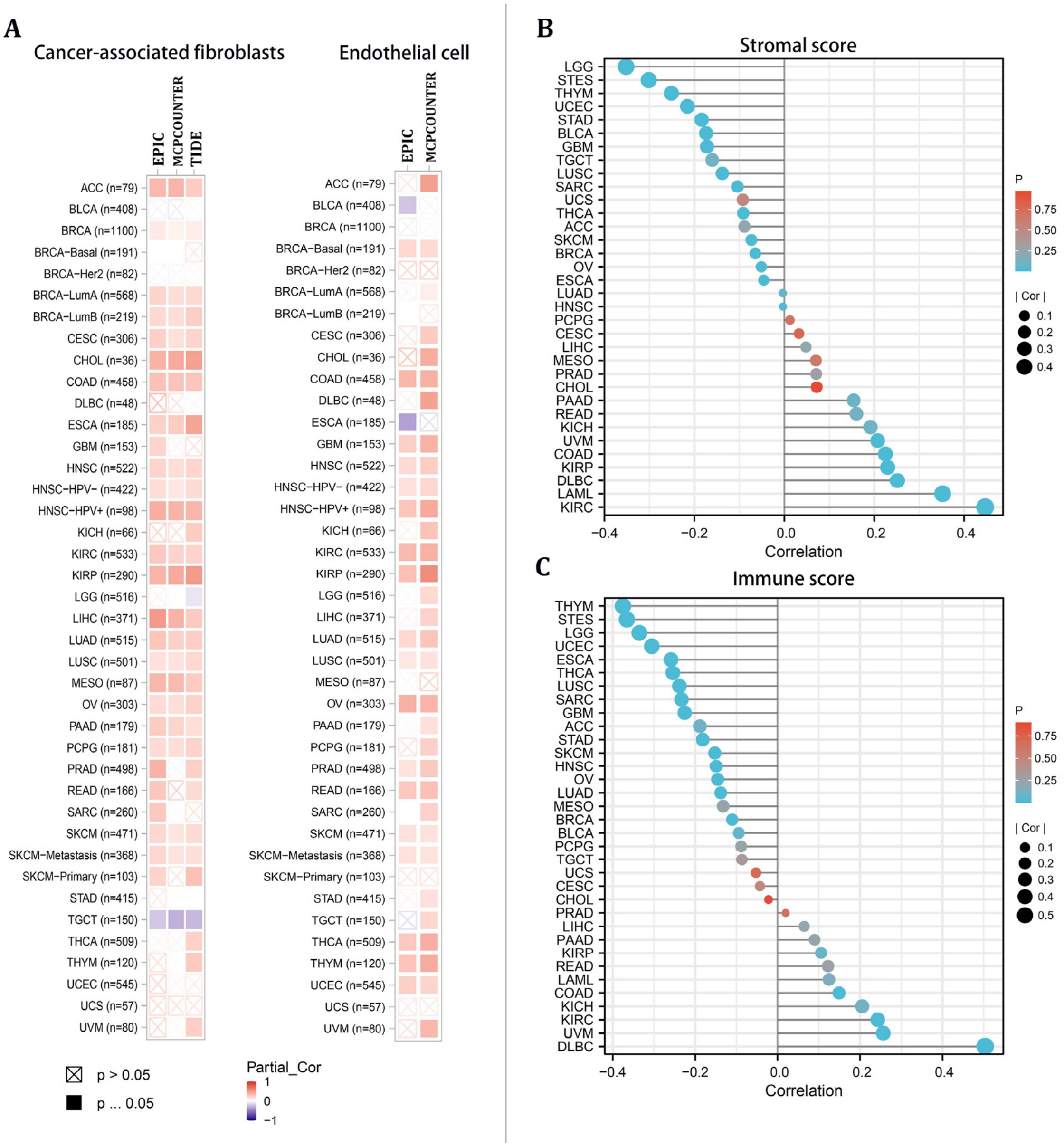

Figure S5: STX6 was positive correlation with Cancer-associated fibroblasts infiltration and Endothelial cell proliferation (**A**). The expression level of STX6 was a robust predictor for tumor stromal score or immune score, especially in LGG, STES, THYM, UCEC, DLBC or KIRC (**B**).
