# Supplementary figures and images for: Syntaxin-6, a Reliable Biomarker for Predicting the Prognosis of Patients with Cancer and the Effectiveness of Immunotherapy
Source: Cancers (Basel). 2022 Dec 21;15(1):27. doi: 10.3390/cancers15010027 (PMC9817965; doi:10.3390/cancers15010027)

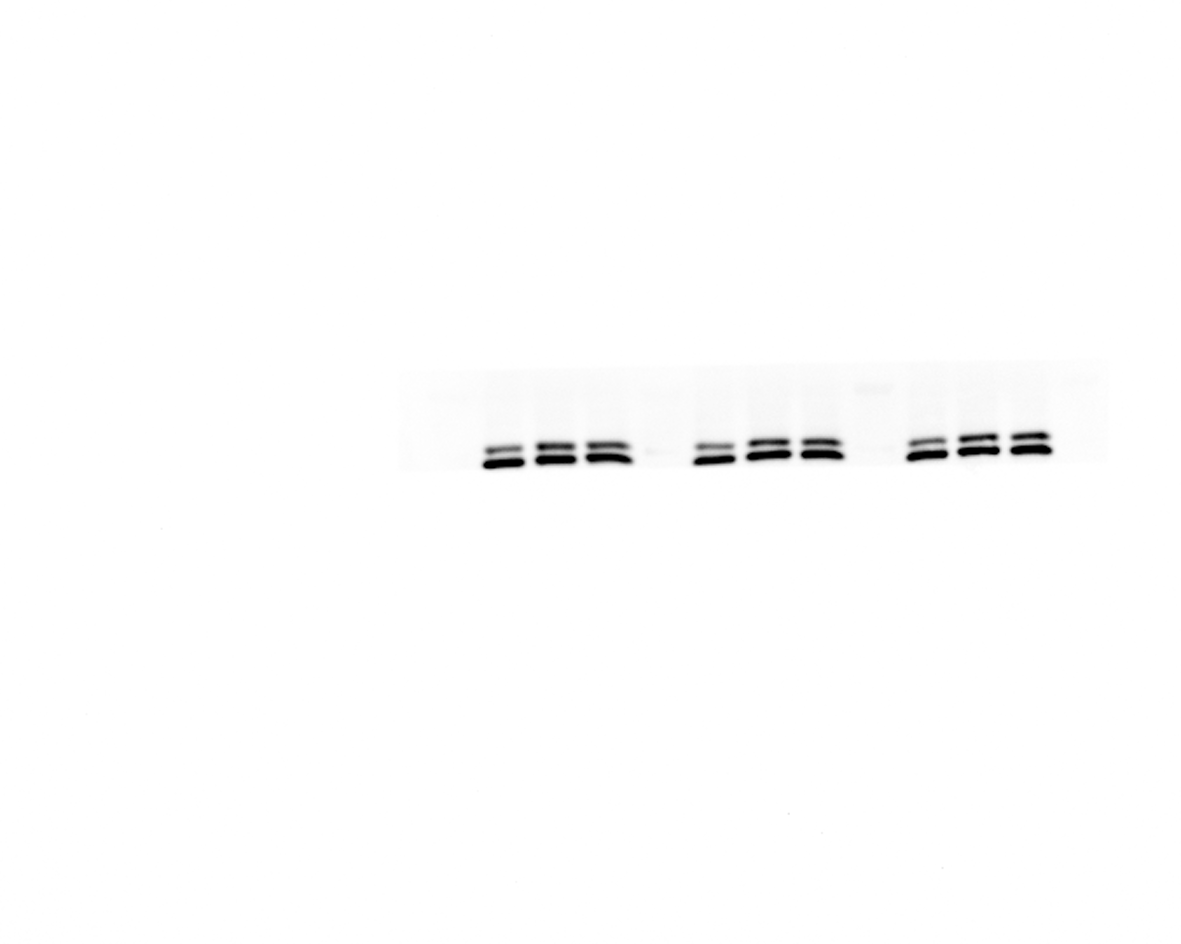

Supplement: Supplementary file 1 [file cancers-15-00027-s001.zip › cancers-2083385-File S2/Figure 8B-8C/B/ERK-shSTX6-BEL7404.tif]

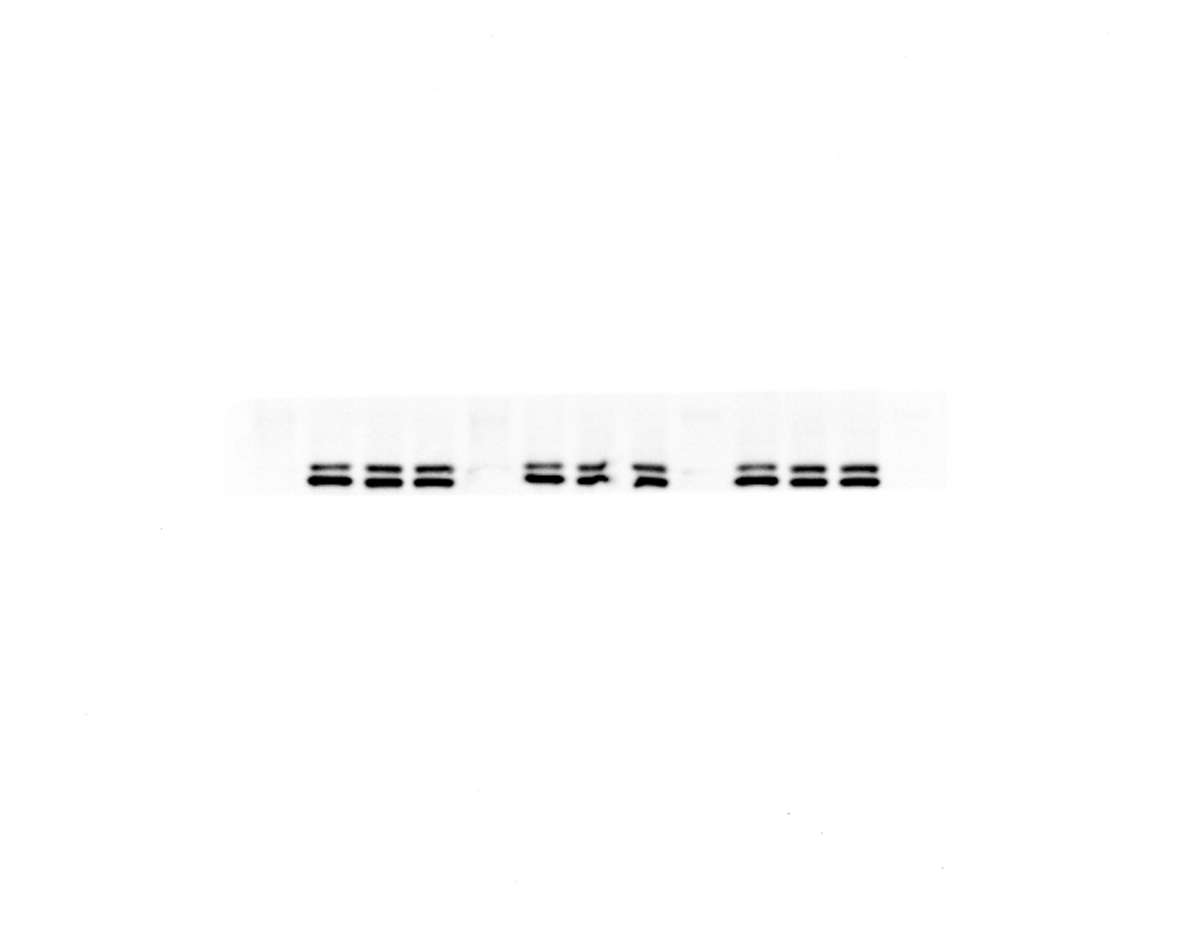

Supplement: Supplementary file 1 [file cancers-15-00027-s001.zip › cancers-2083385-File S2/Figure 8B-8C/B/ERK-shSTX6-hct116.tif]

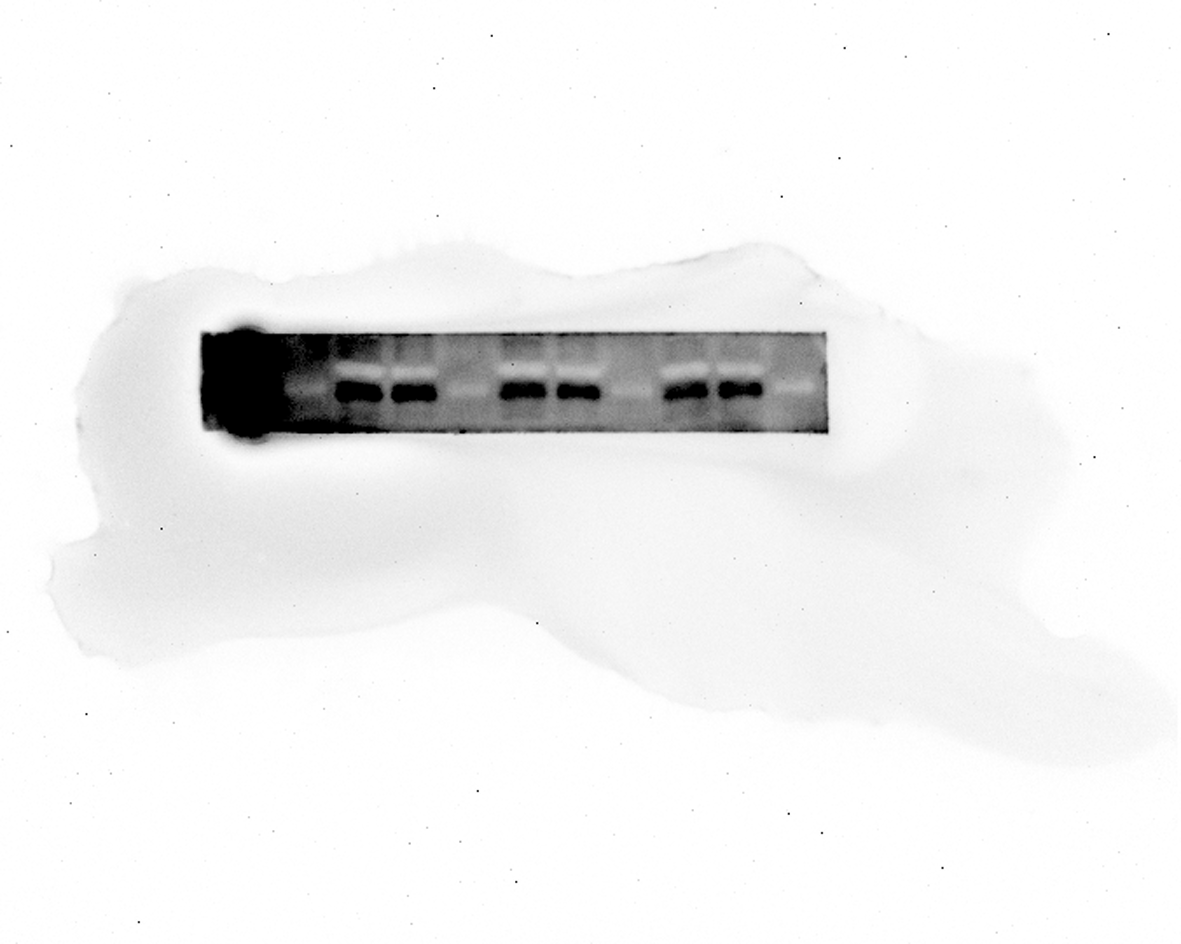

Supplement: Supplementary file 1 [file cancers-15-00027-s001.zip › cancers-2083385-File S2/Figure 8B-8C/B/ERK-SW480-OE.tif]

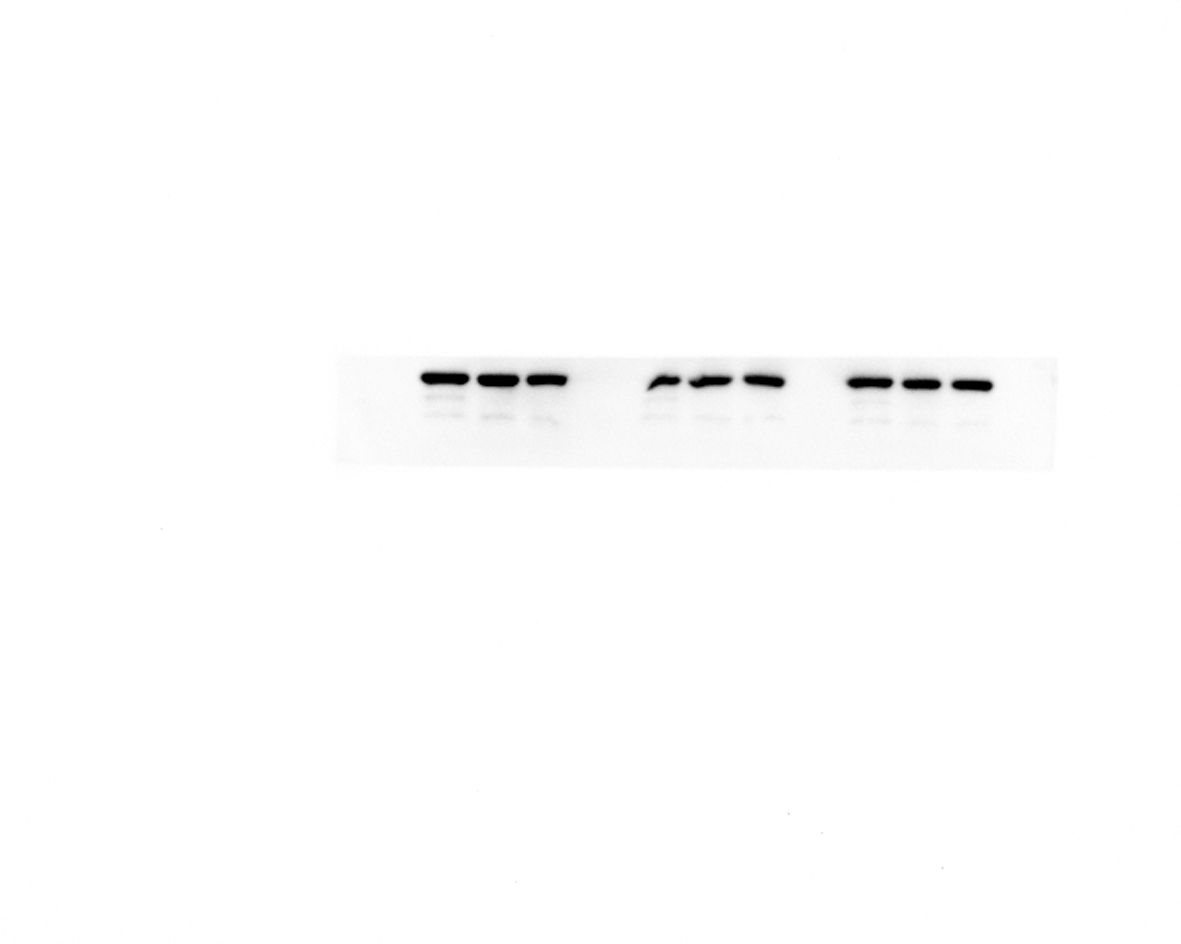

Supplement: Supplementary file 1 [file cancers-15-00027-s001.zip › cancers-2083385-File S2/Figure 8B-8C/B/GAPDH-shSTX6-BEL7404.tif]

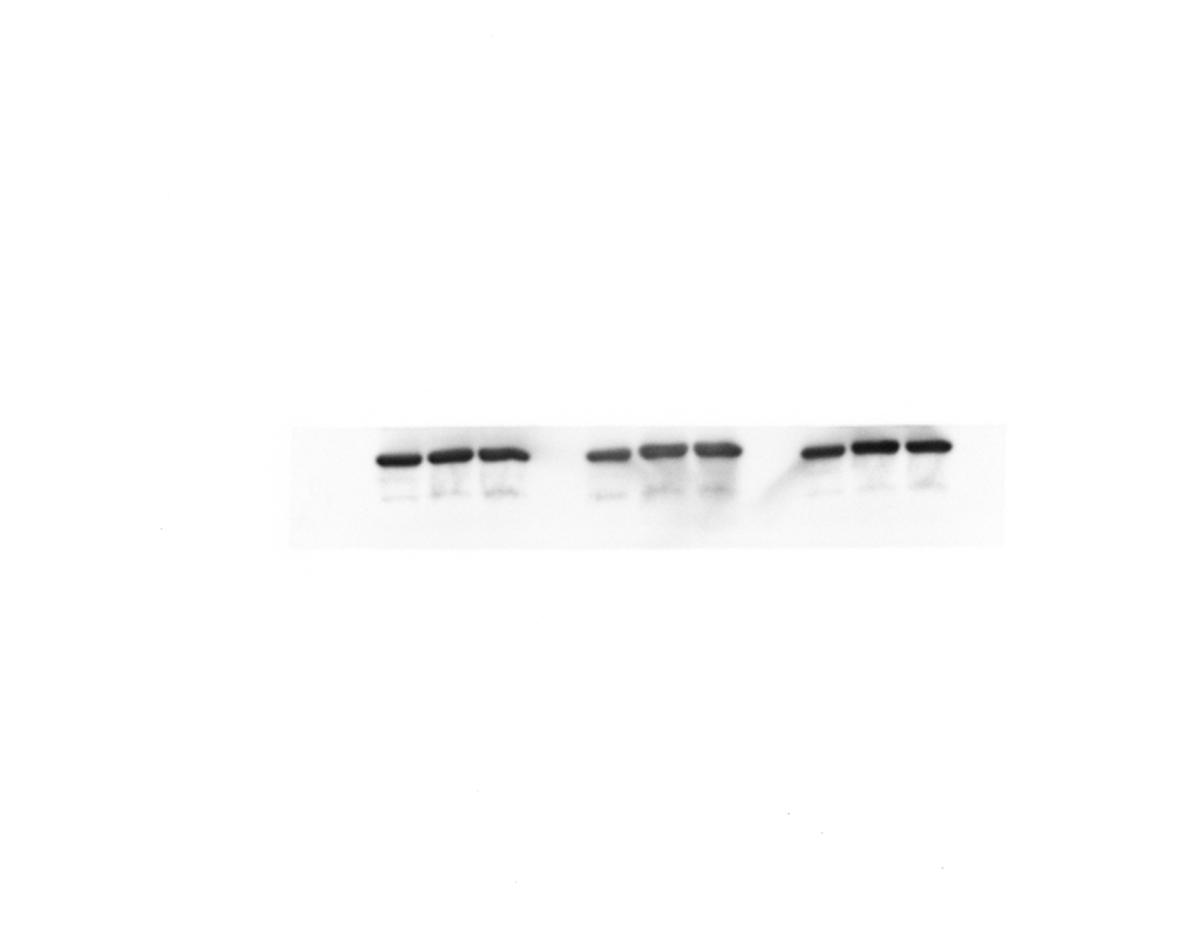

Supplement: Supplementary file 1 [file cancers-15-00027-s001.zip › cancers-2083385-File S2/Figure 8B-8C/B/GAPDH-shSTX6-HCT116.tif]

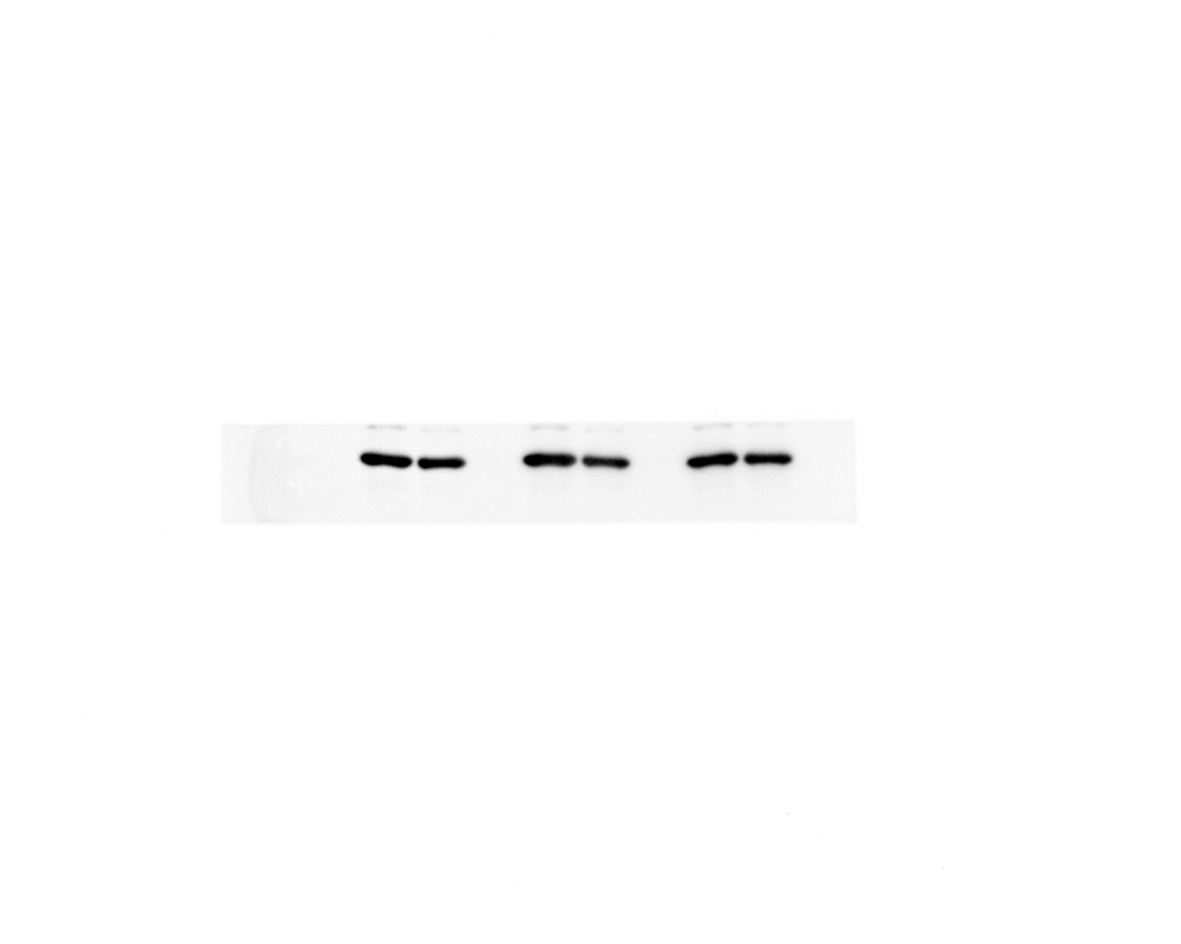

Supplement: Supplementary file 1 [file cancers-15-00027-s001.zip › cancers-2083385-File S2/Figure 8B-8C/B/GAPDH-SW480-OE.tif]

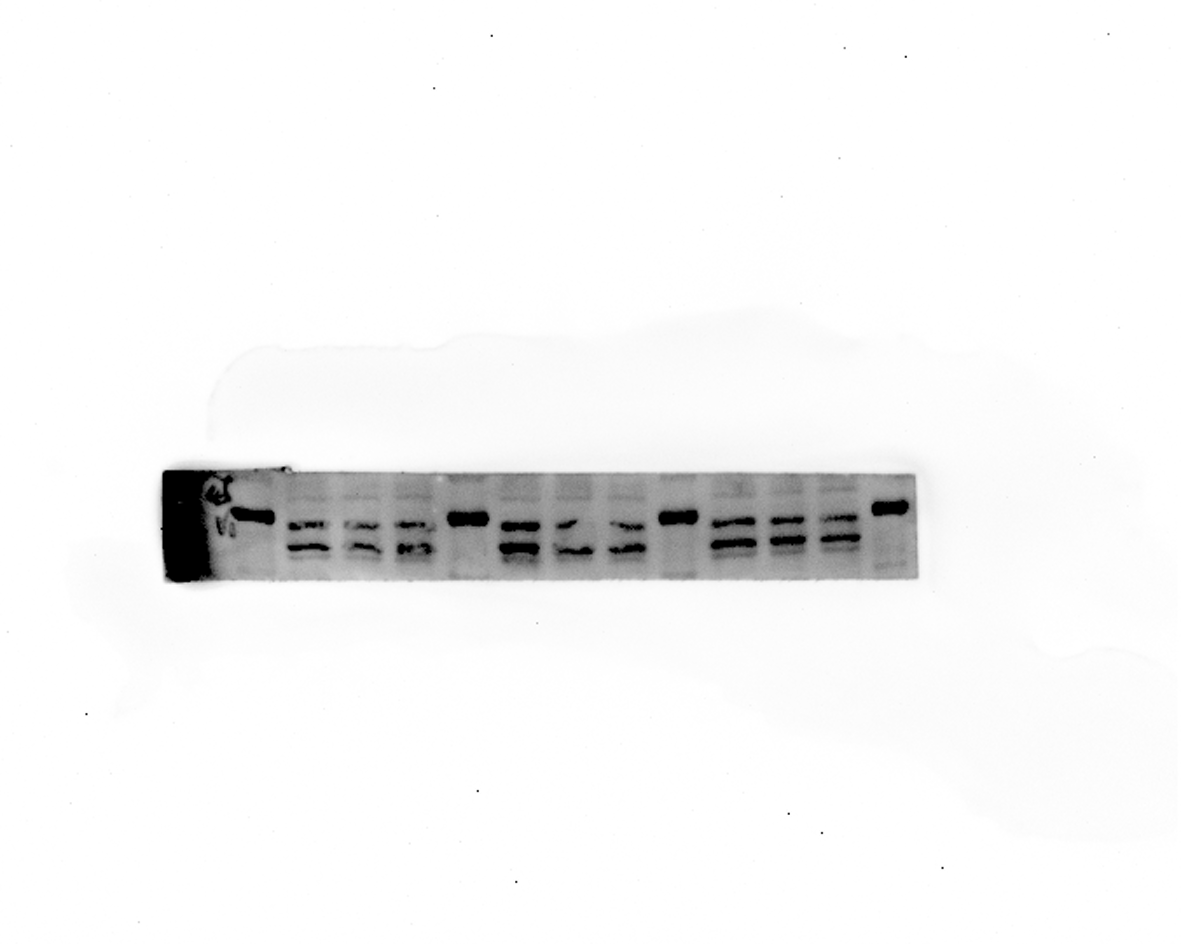

Supplement: Supplementary file 1 [file cancers-15-00027-s001.zip › cancers-2083385-File S2/Figure 8B-8C/B/MRK-shSTX6-BEL7404.tif]

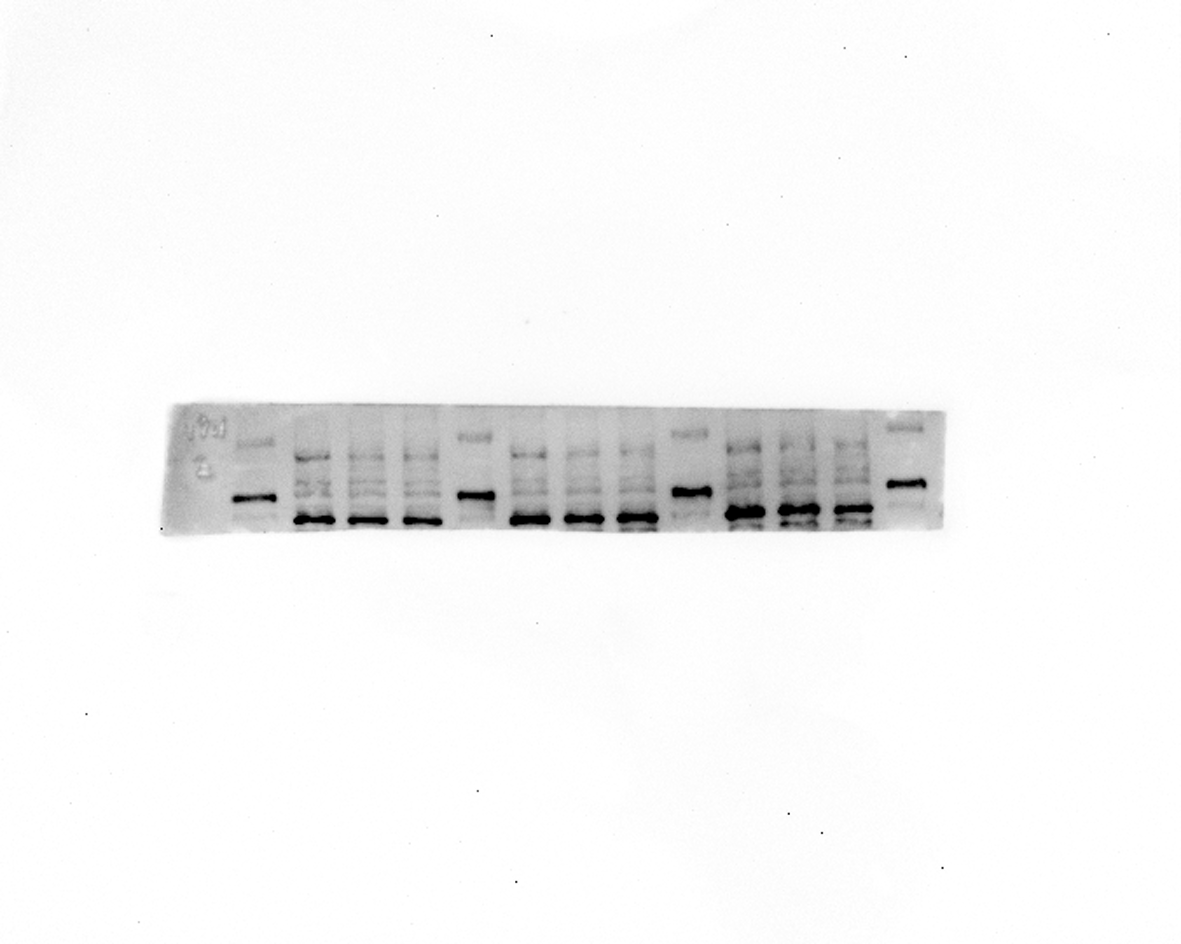

Supplement: Supplementary file 1 [file cancers-15-00027-s001.zip › cancers-2083385-File S2/Figure 8B-8C/B/MRK-shSTX6-HCT116.tif]

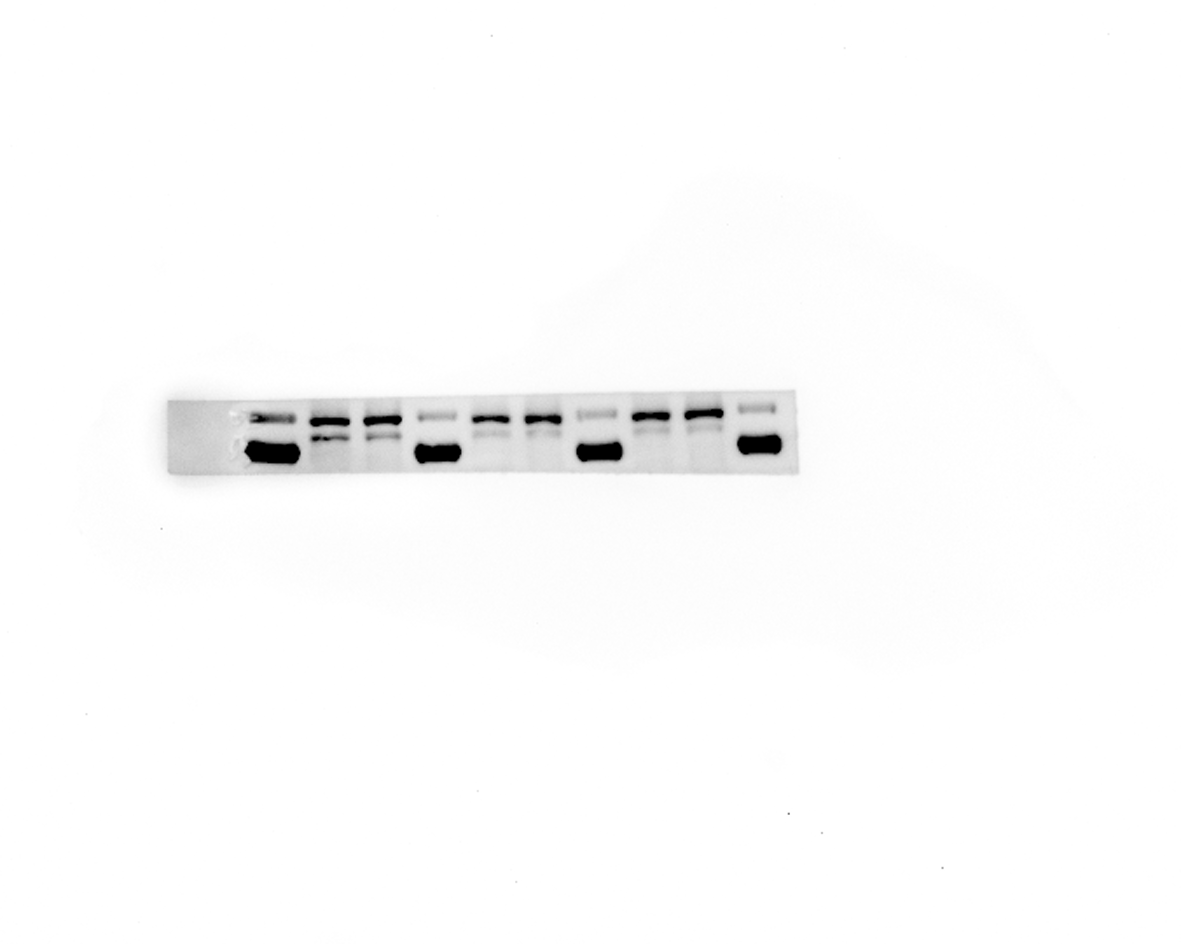

Supplement: Supplementary file 1 [file cancers-15-00027-s001.zip › cancers-2083385-File S2/Figure 8B-8C/B/MRK-SW480-OE.tif]

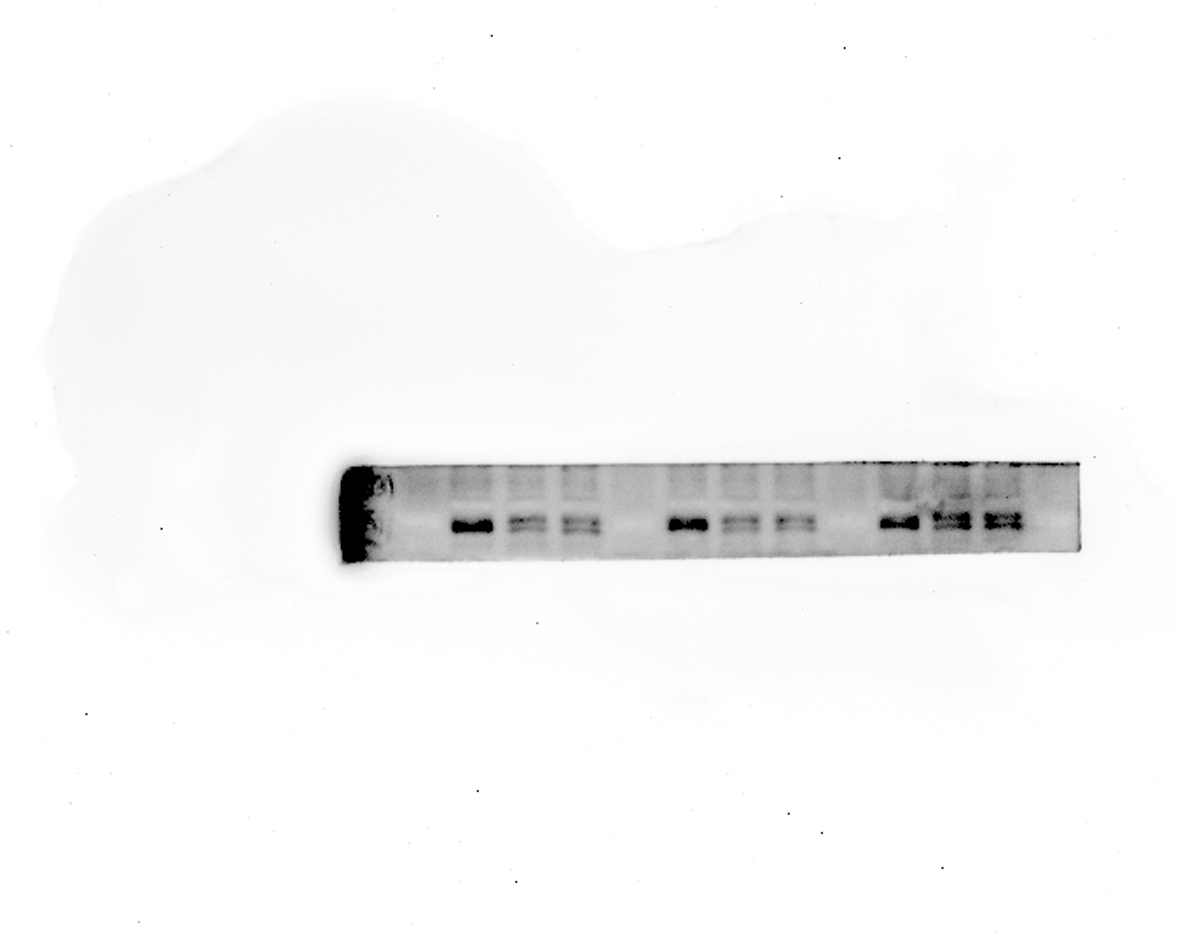

Supplement: Supplementary file 1 [file cancers-15-00027-s001.zip › cancers-2083385-File S2/Figure 8B-8C/B/P-ERK-shSTX6-BEL7404.tif]

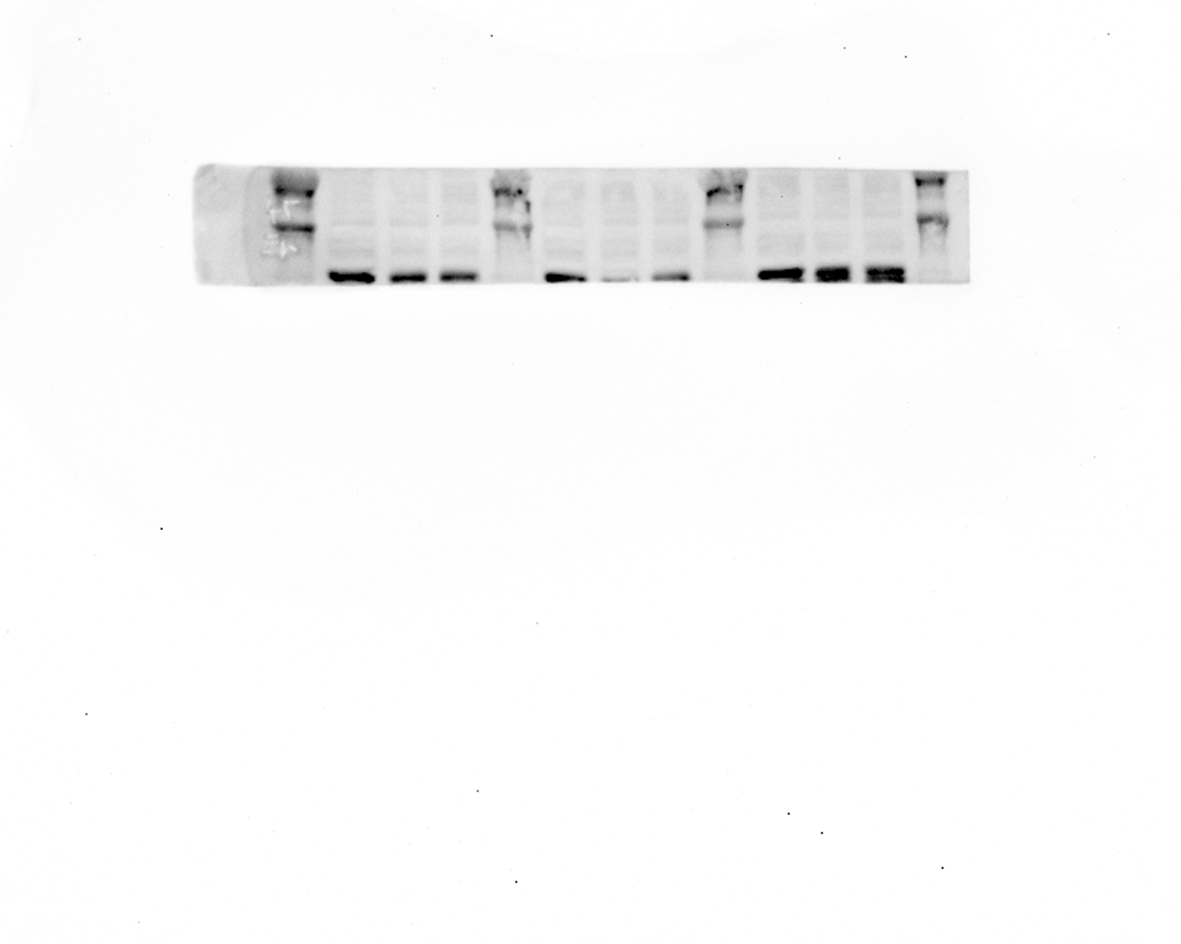

Supplement: Supplementary file 1 [file cancers-15-00027-s001.zip › cancers-2083385-File S2/Figure 8B-8C/B/p-ERK-shSTX6-HCT116.tif]

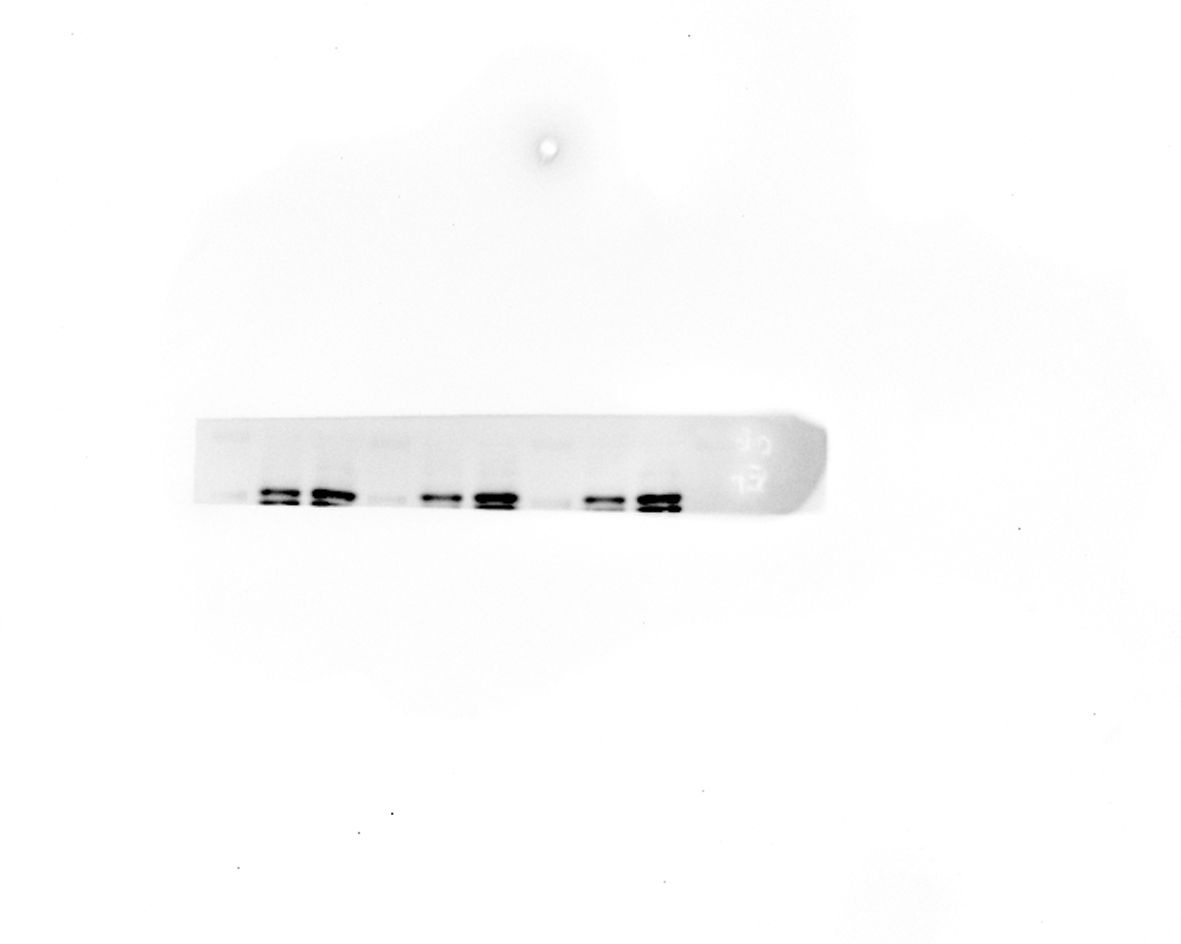

Supplement: Supplementary file 1 [file cancers-15-00027-s001.zip › cancers-2083385-File S2/Figure 8B-8C/B/p-erk-SW480-OE.tif]

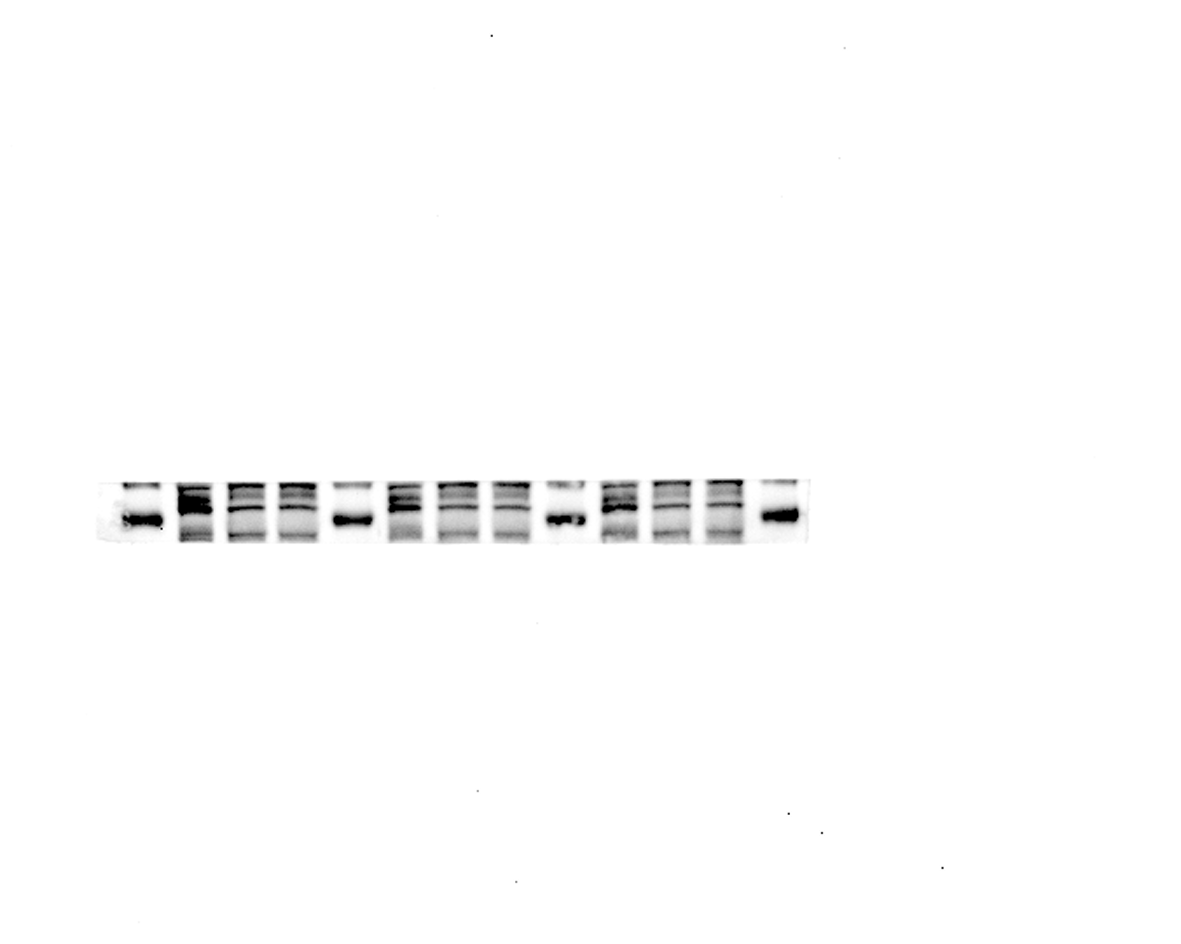

Supplement: Supplementary file 1 [file cancers-15-00027-s001.zip › cancers-2083385-File S2/Figure 8B-8C/B/p-MEK-shSTX6-BEL7404.tif]

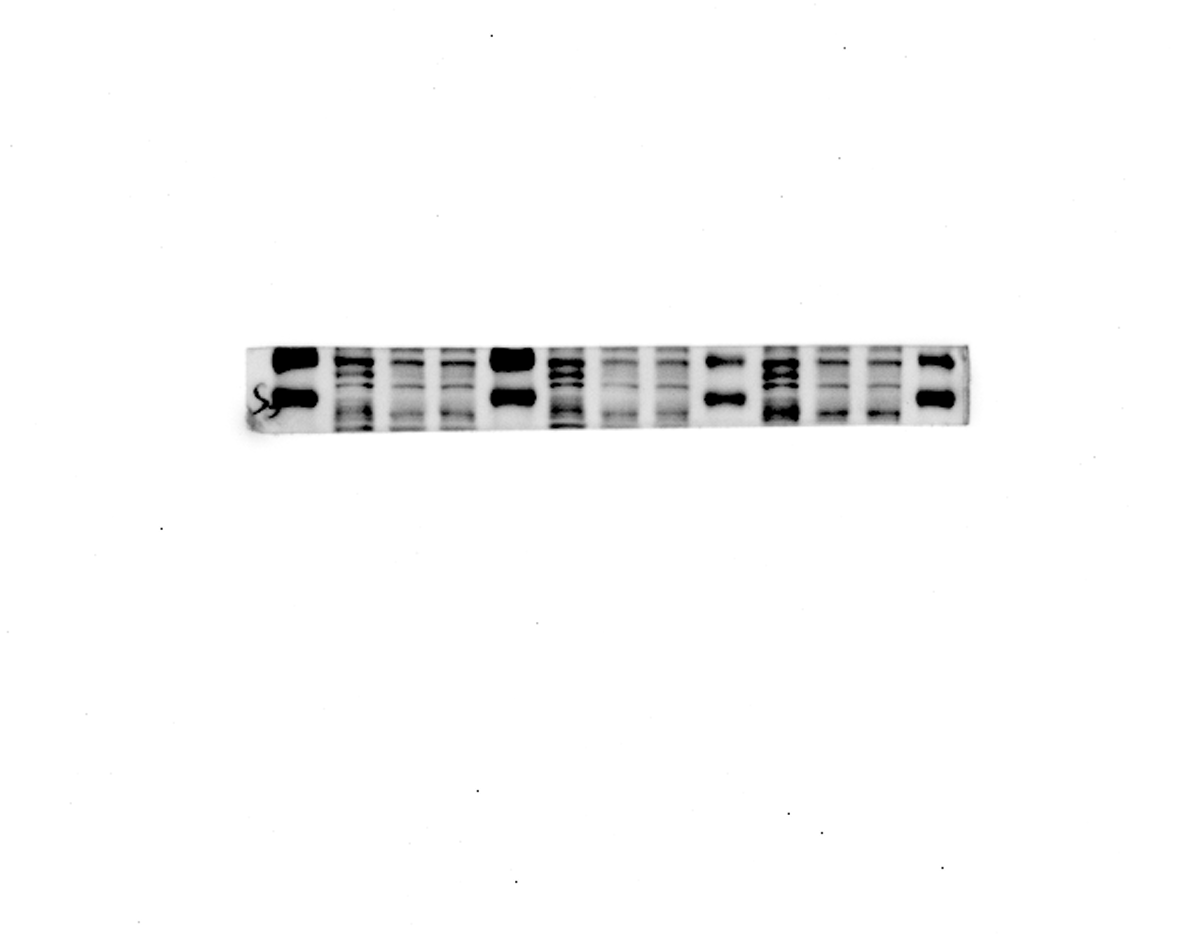

Supplement: Supplementary file 1 [file cancers-15-00027-s001.zip › cancers-2083385-File S2/Figure 8B-8C/B/p-MEK-shSTX6-HCT116.tif]

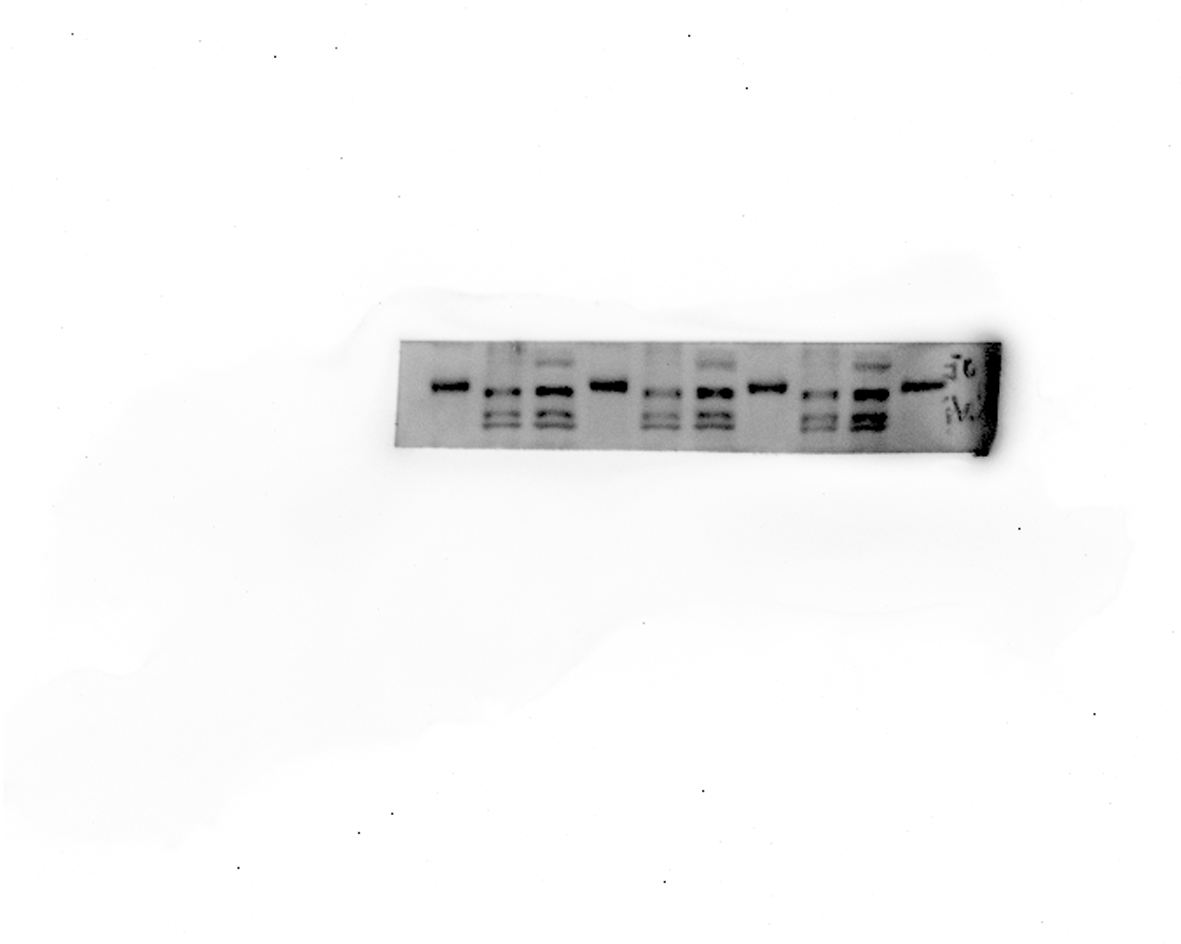

Supplement: Supplementary file 1 [file cancers-15-00027-s001.zip › cancers-2083385-File S2/Figure 8B-8C/B/p-mrk-sw480-oe.tif]

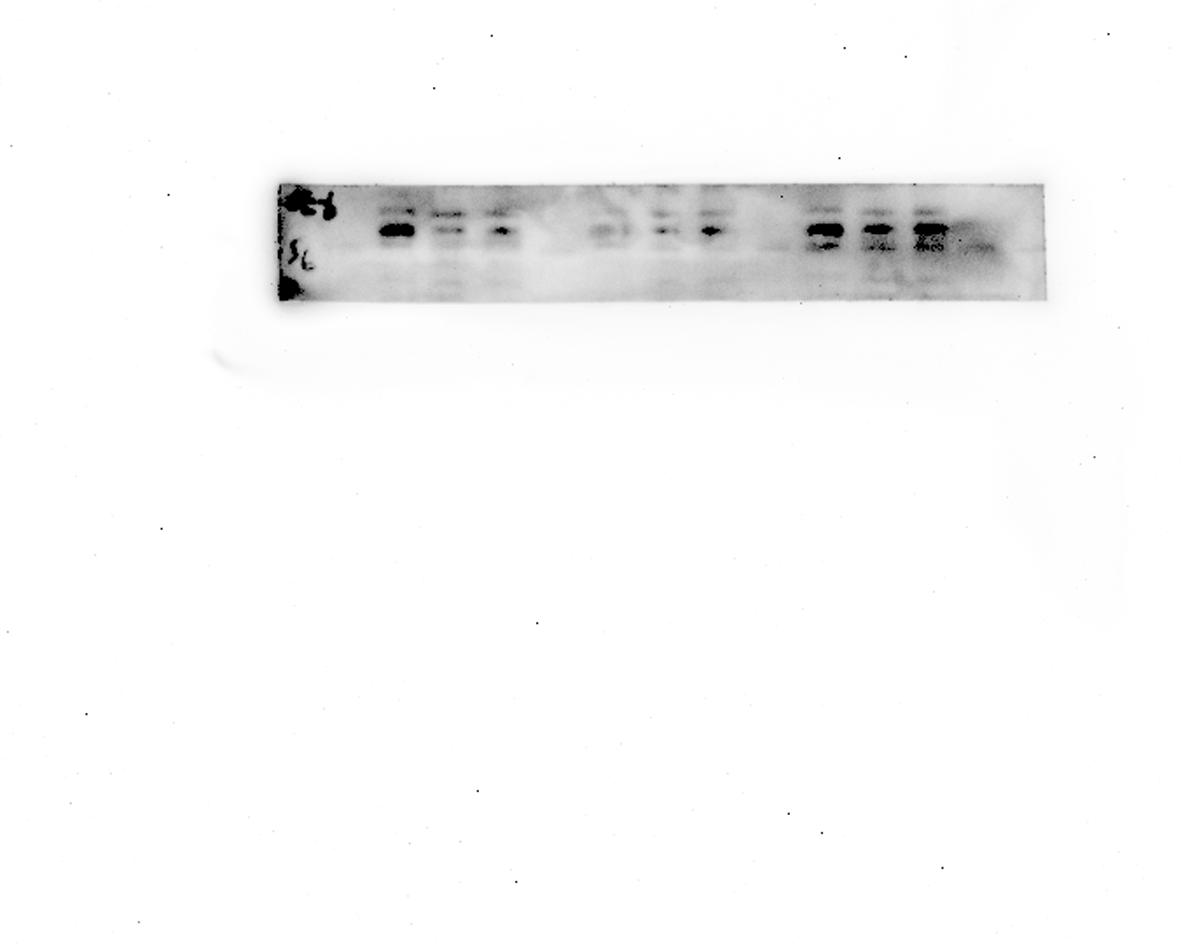

Supplement: Supplementary file 1 [file cancers-15-00027-s001.zip › cancers-2083385-File S2/Figure 8B-8C/B/stx6-sh-BEL7404.tif]

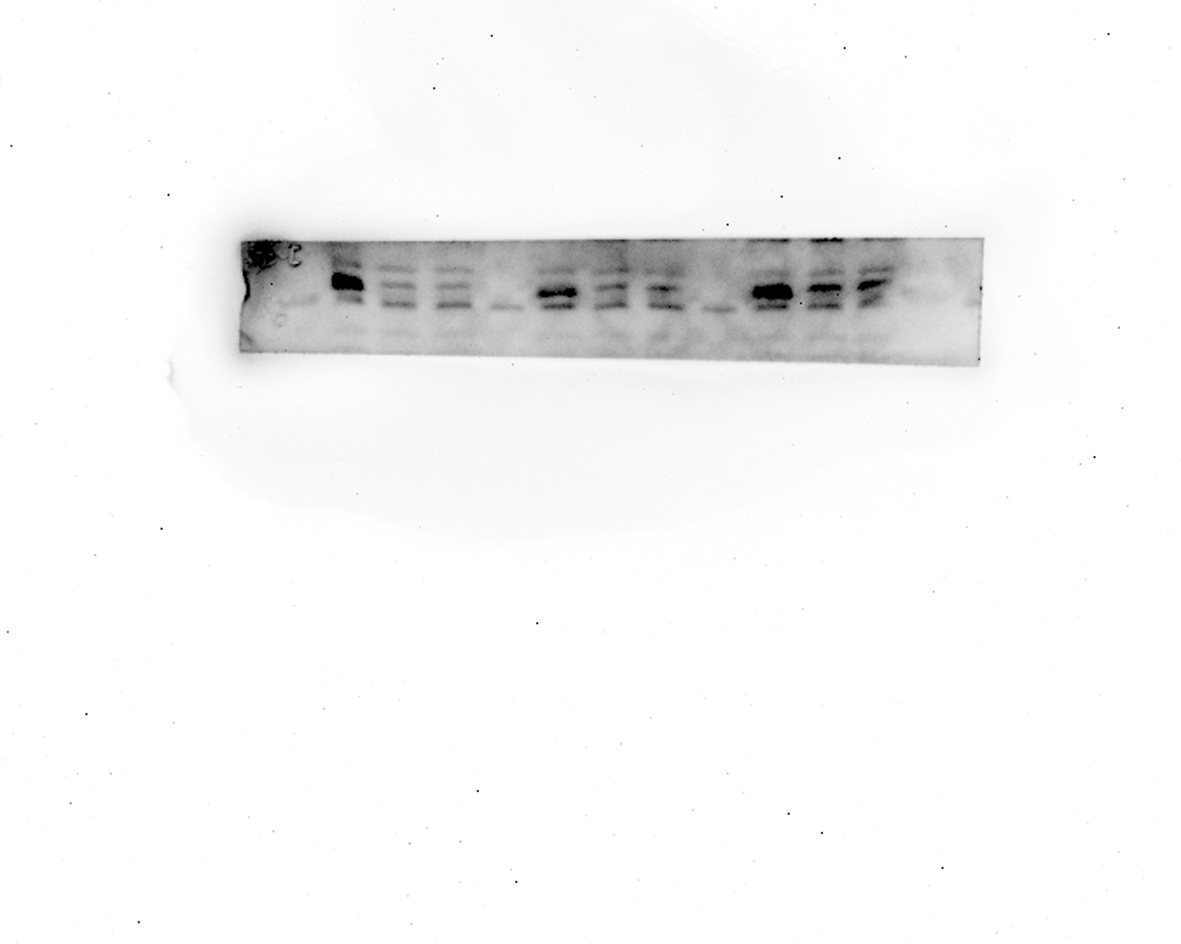

Supplement: Supplementary file 1 [file cancers-15-00027-s001.zip › cancers-2083385-File S2/Figure 8B-8C/B/stx6-sh-HCT116.tif]

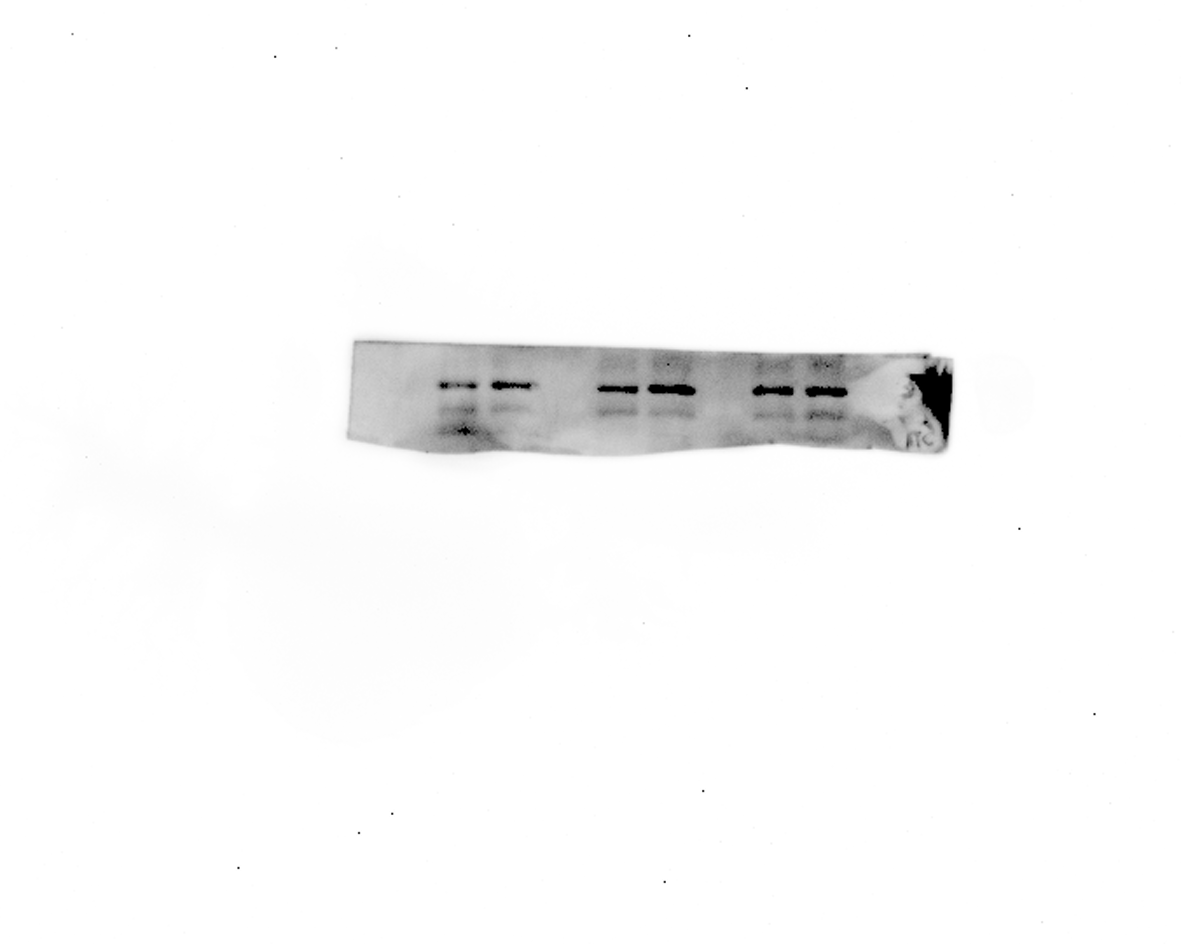

Supplement: Supplementary file 1 [file cancers-15-00027-s001.zip › cancers-2083385-File S2/Figure 8B-8C/B/stx6-SW480-OE.tif]

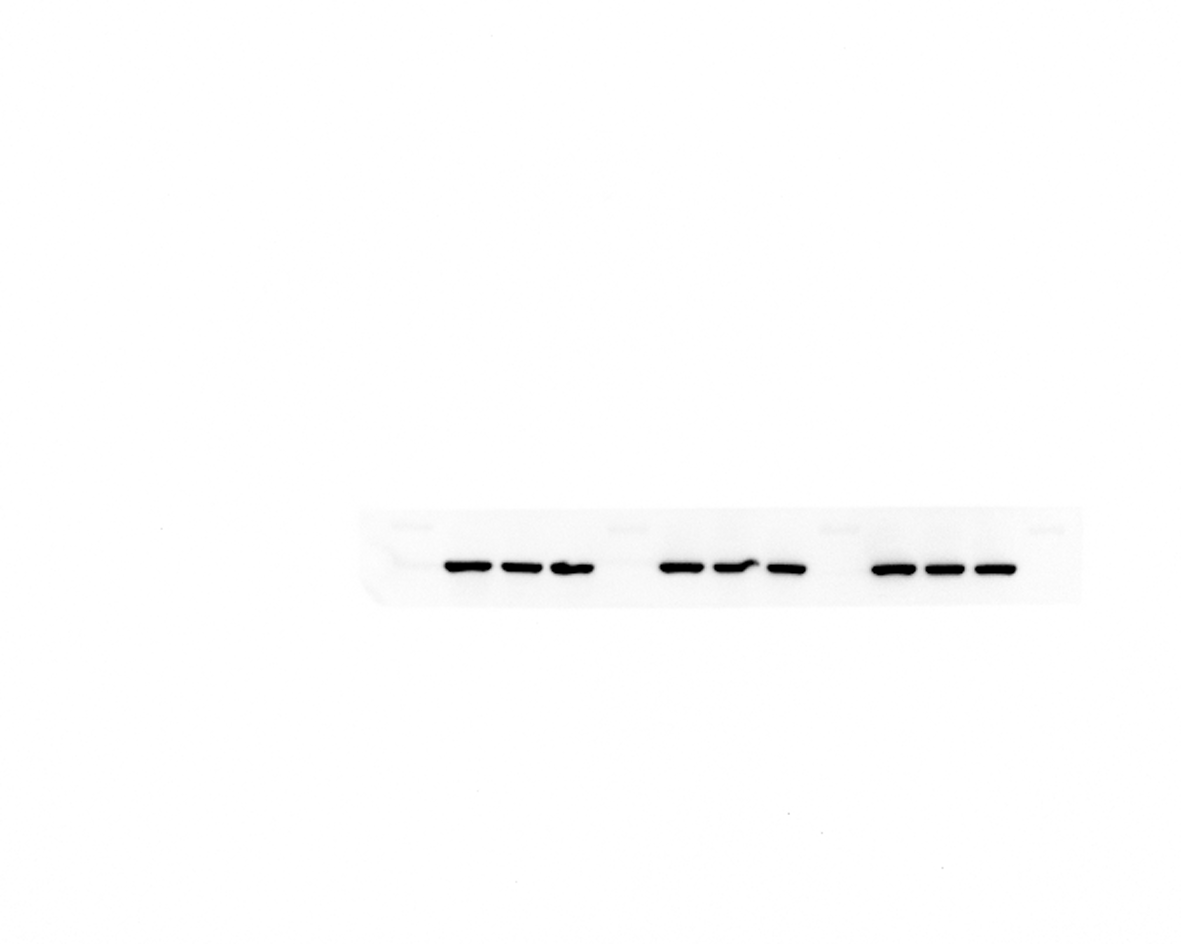

Supplement: Supplementary file 1 [file cancers-15-00027-s001.zip › cancers-2083385-File S2/Figure 8B-8C/C/Actin-HCT116-shSTX6.tif]

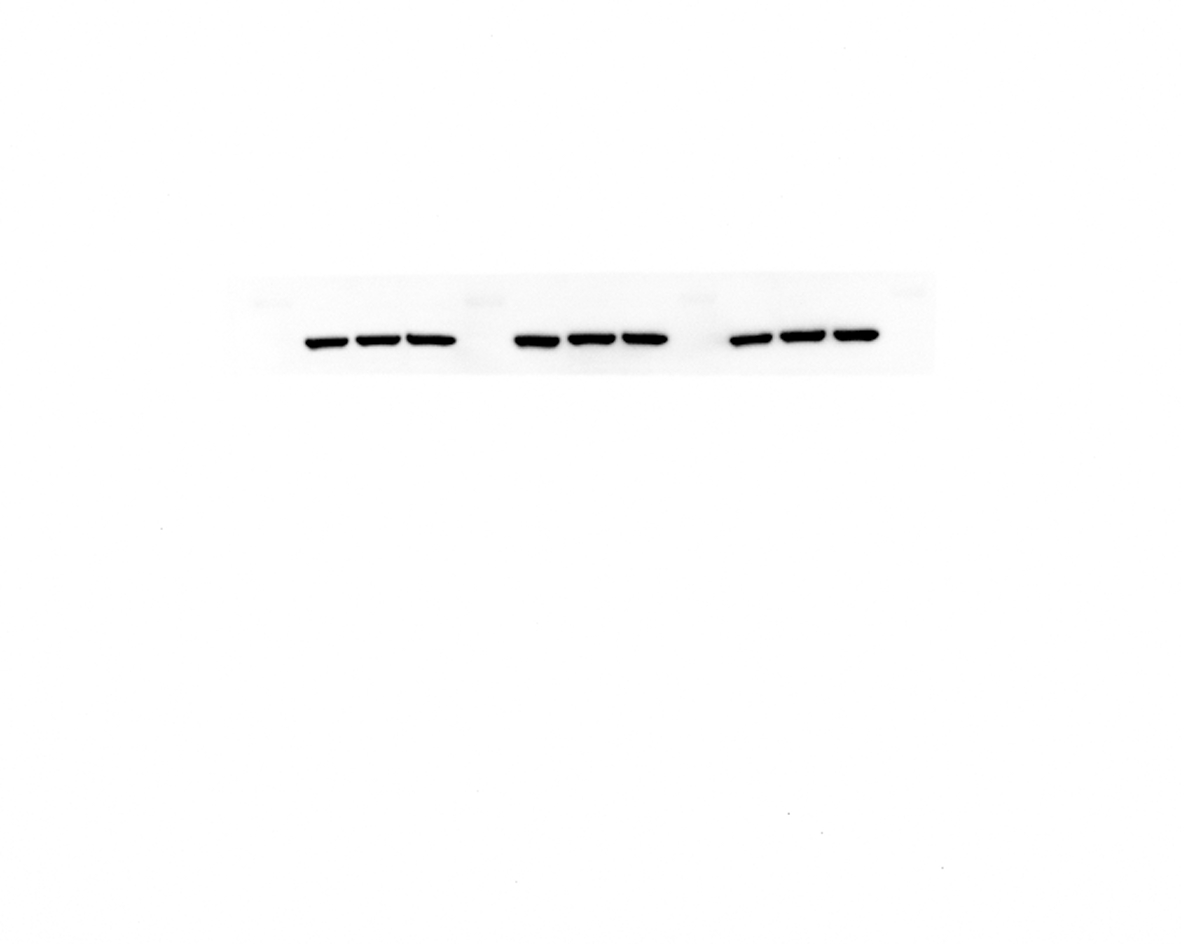

Supplement: Supplementary file 1 [file cancers-15-00027-s001.zip › cancers-2083385-File S2/Figure 8B-8C/C/ACTIN-shSTX6-BEL-7404.tif]

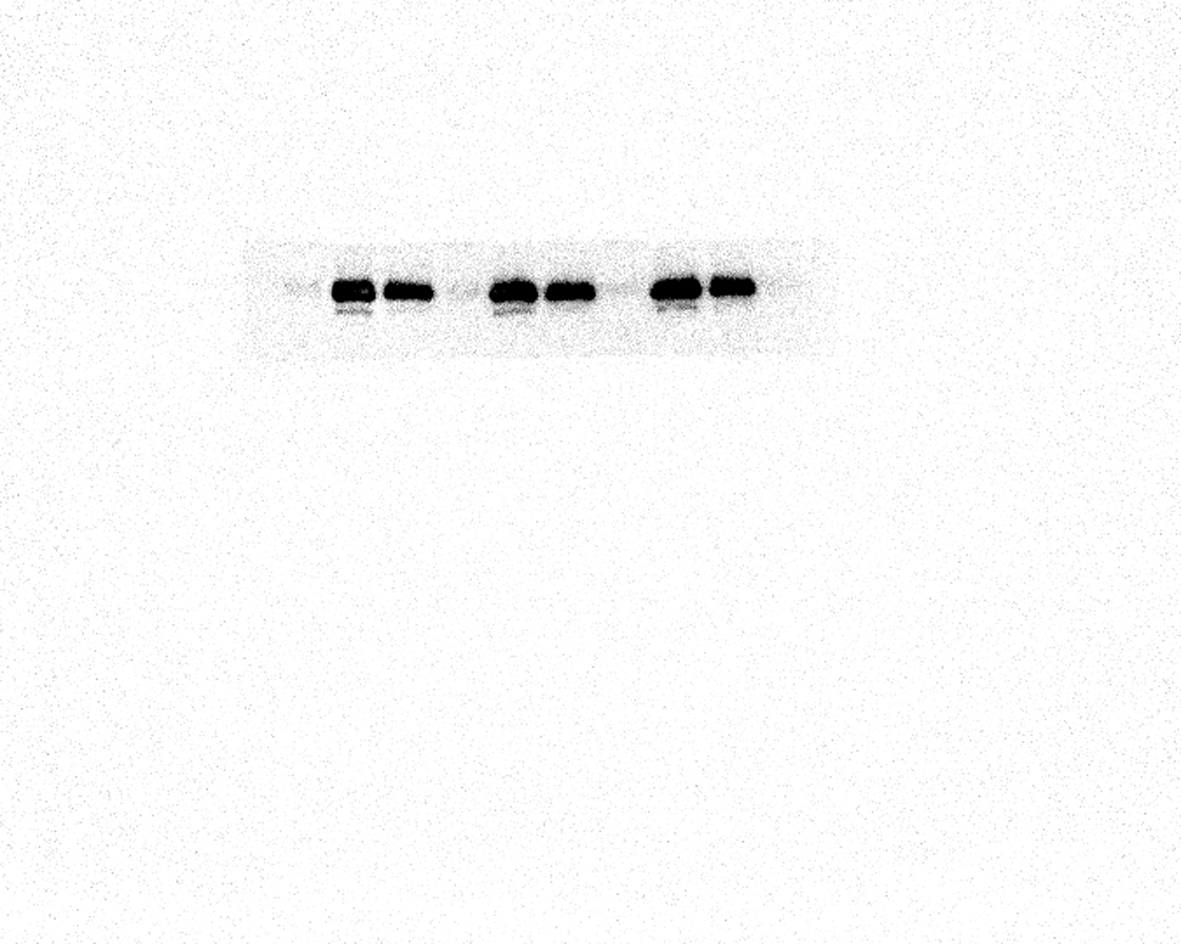

Supplement: Supplementary file 1 [file cancers-15-00027-s001.zip › cancers-2083385-File S2/Figure 8B-8C/C/Actin-SW480-OE.tif]

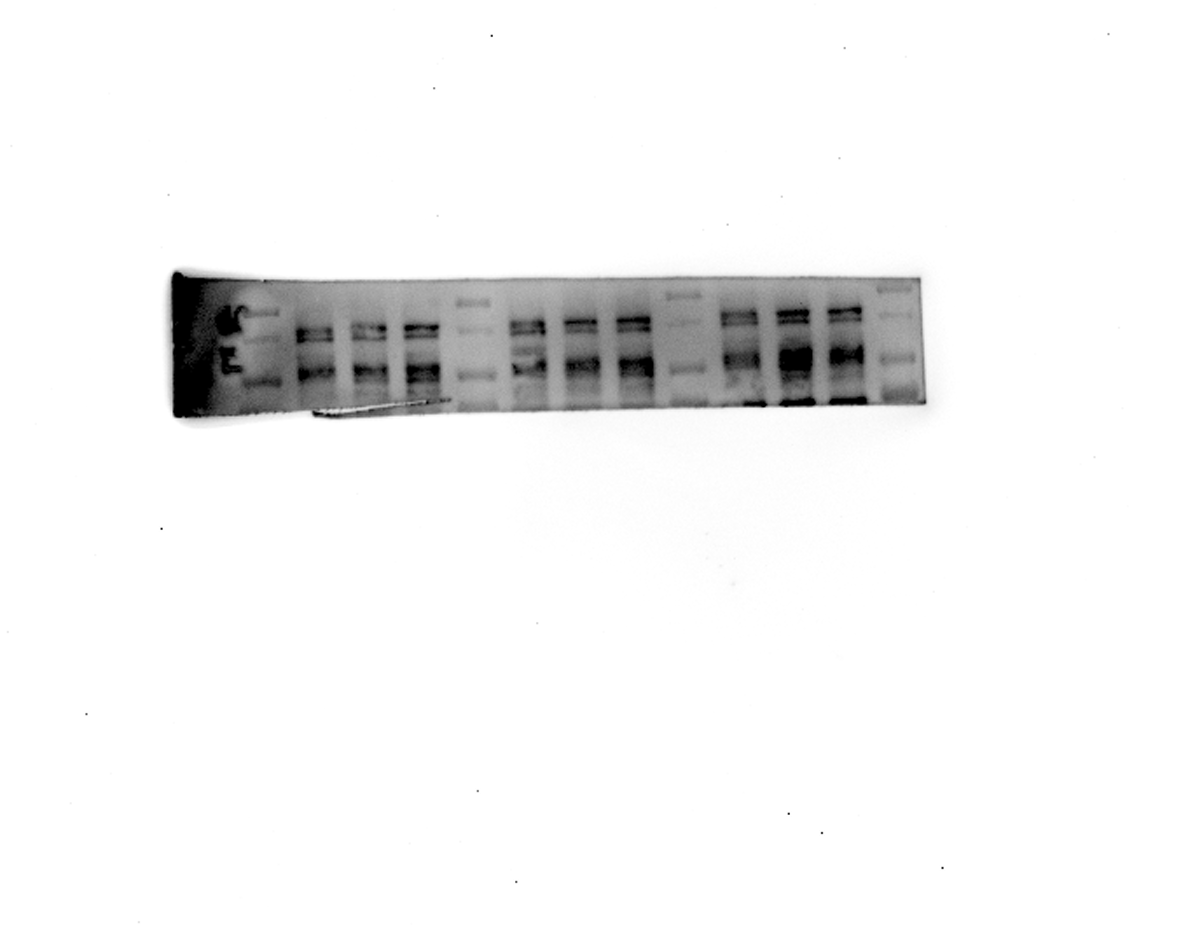

Supplement: Supplementary file 1 [file cancers-15-00027-s001.zip › cancers-2083385-File S2/Figure 8B-8C/C/Ecadherin-BEL-7404-shSTX6.tif]

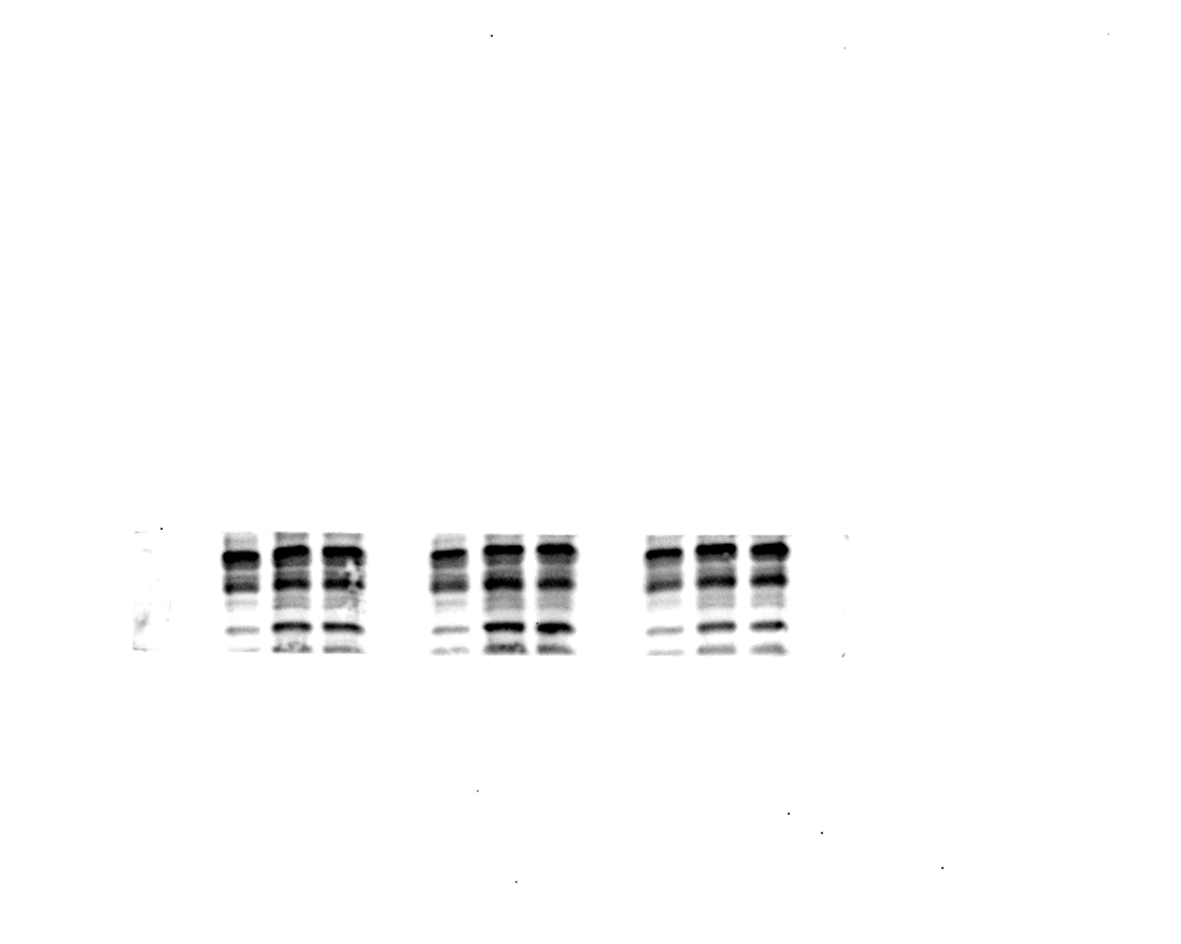

Supplement: Supplementary file 1 [file cancers-15-00027-s001.zip › cancers-2083385-File S2/Figure 8B-8C/C/Ecadherin-shSTX6-HCT116.tif]

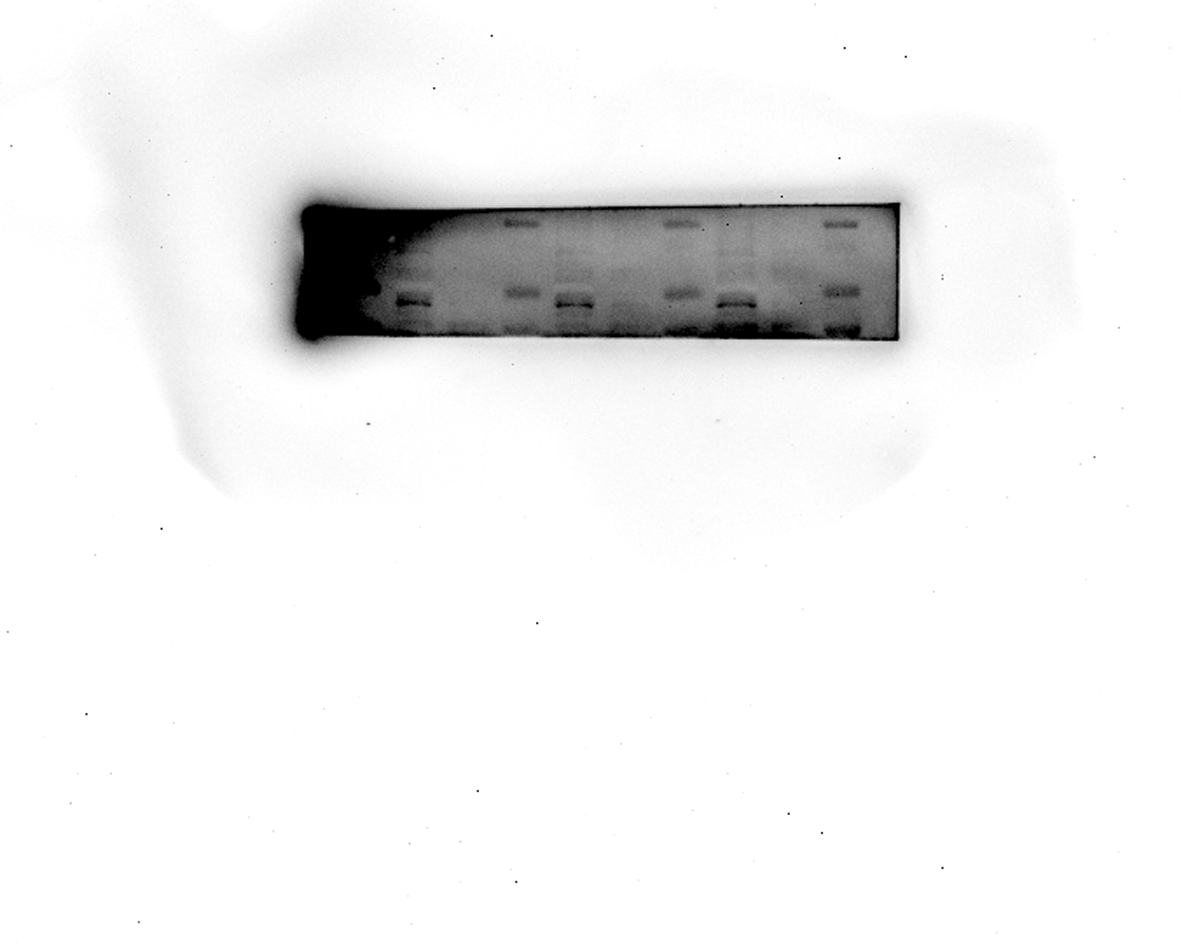

Supplement: Supplementary file 1 [file cancers-15-00027-s001.zip › cancers-2083385-File S2/Figure 8B-8C/C/Ecadherin-SW480-OE.tif]

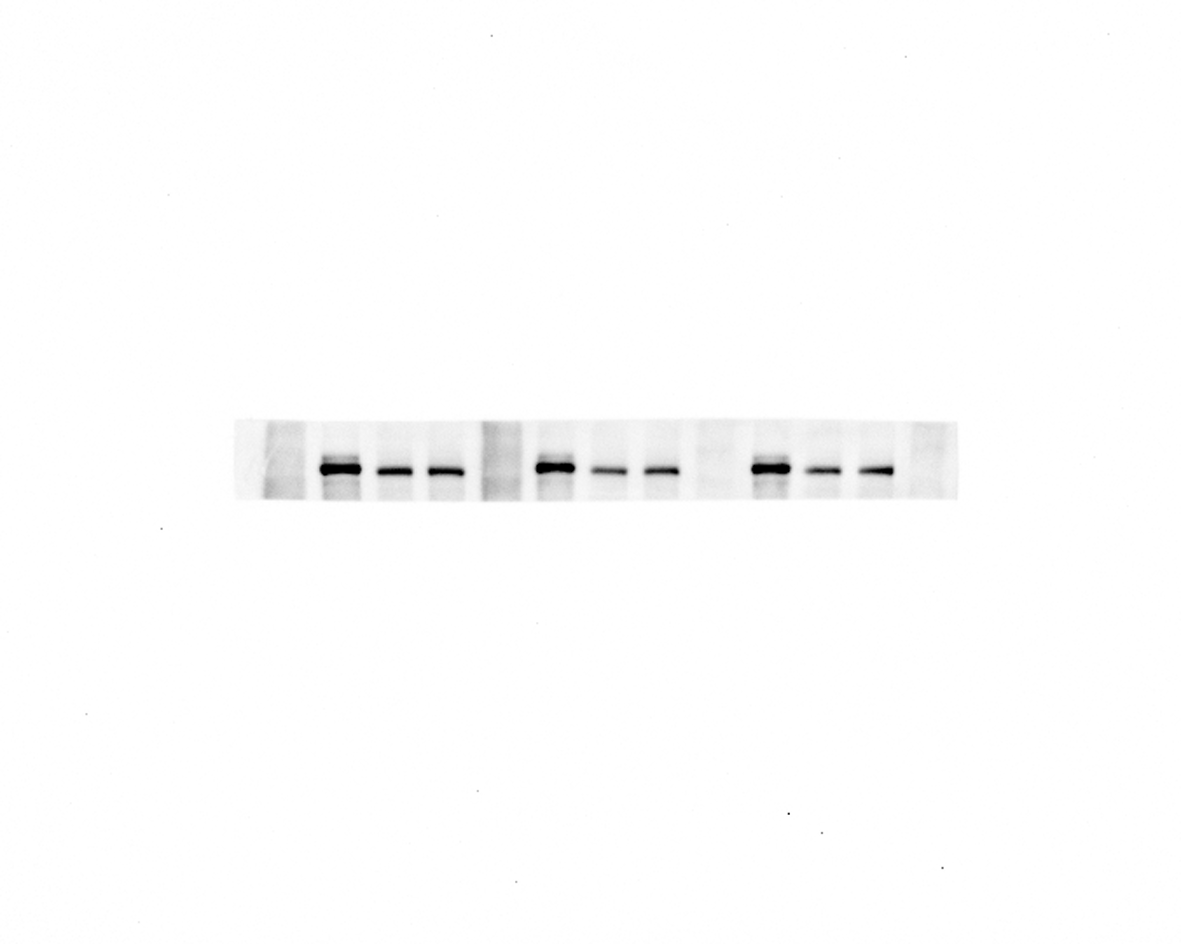

Supplement: Supplementary file 1 [file cancers-15-00027-s001.zip › cancers-2083385-File S2/Figure 8B-8C/C/Ncadherin-BEL7404-shSTX6.tif]

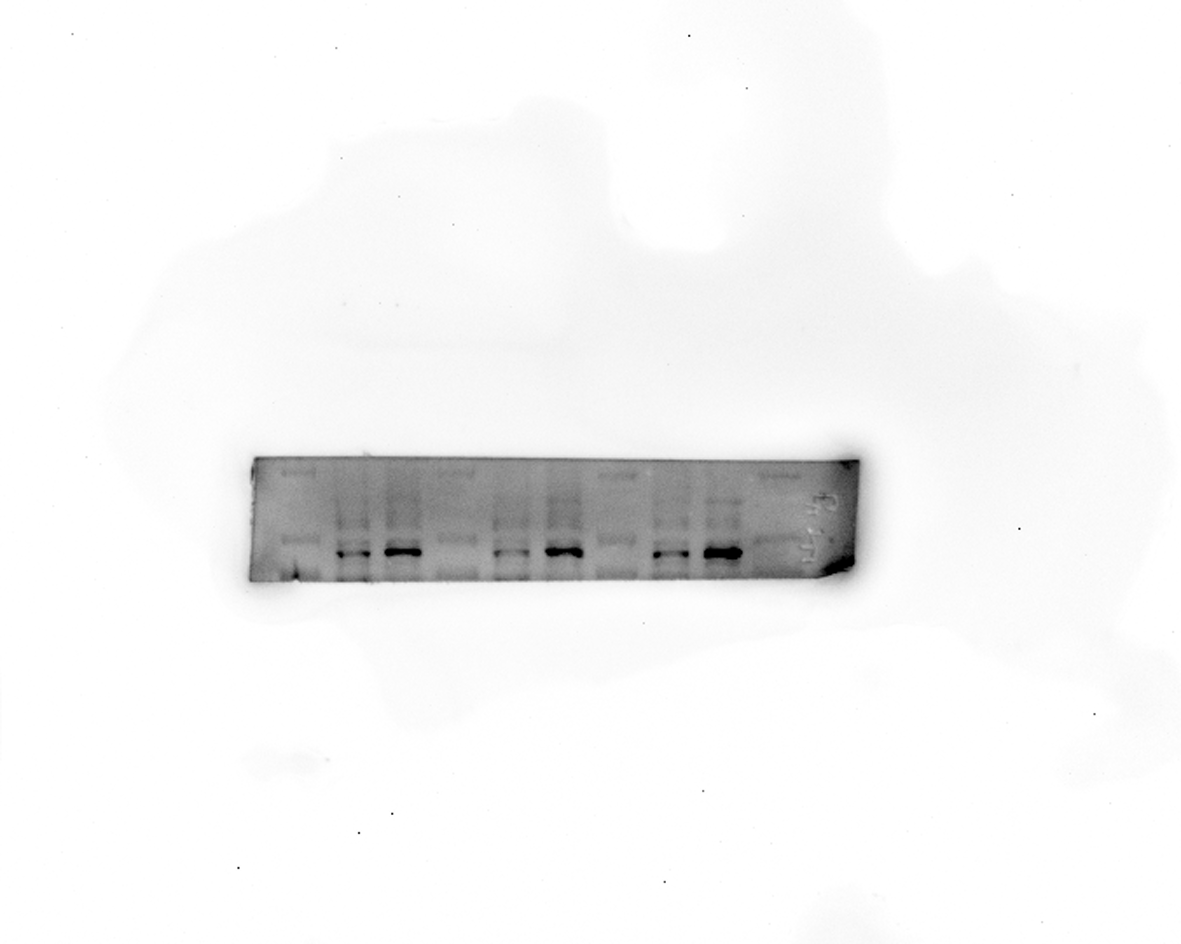

Supplement: Supplementary file 1 [file cancers-15-00027-s001.zip › cancers-2083385-File S2/Figure 8B-8C/C/Ncadherin-sw480-OE.tif]

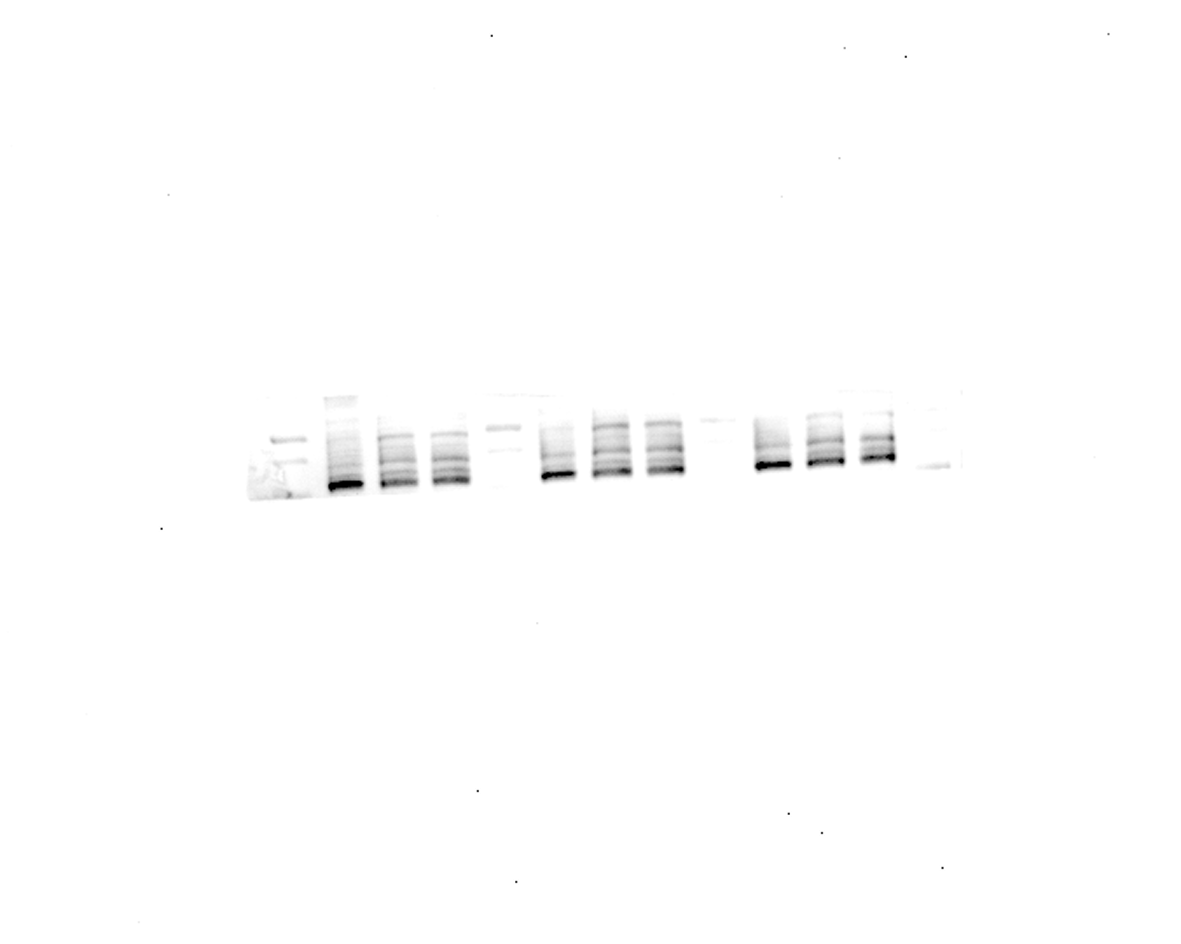

Supplement: Supplementary file 1 [file cancers-15-00027-s001.zip › cancers-2083385-File S2/Figure 8B-8C/C/Ncadherin_HCT116-shSTX6.tif]

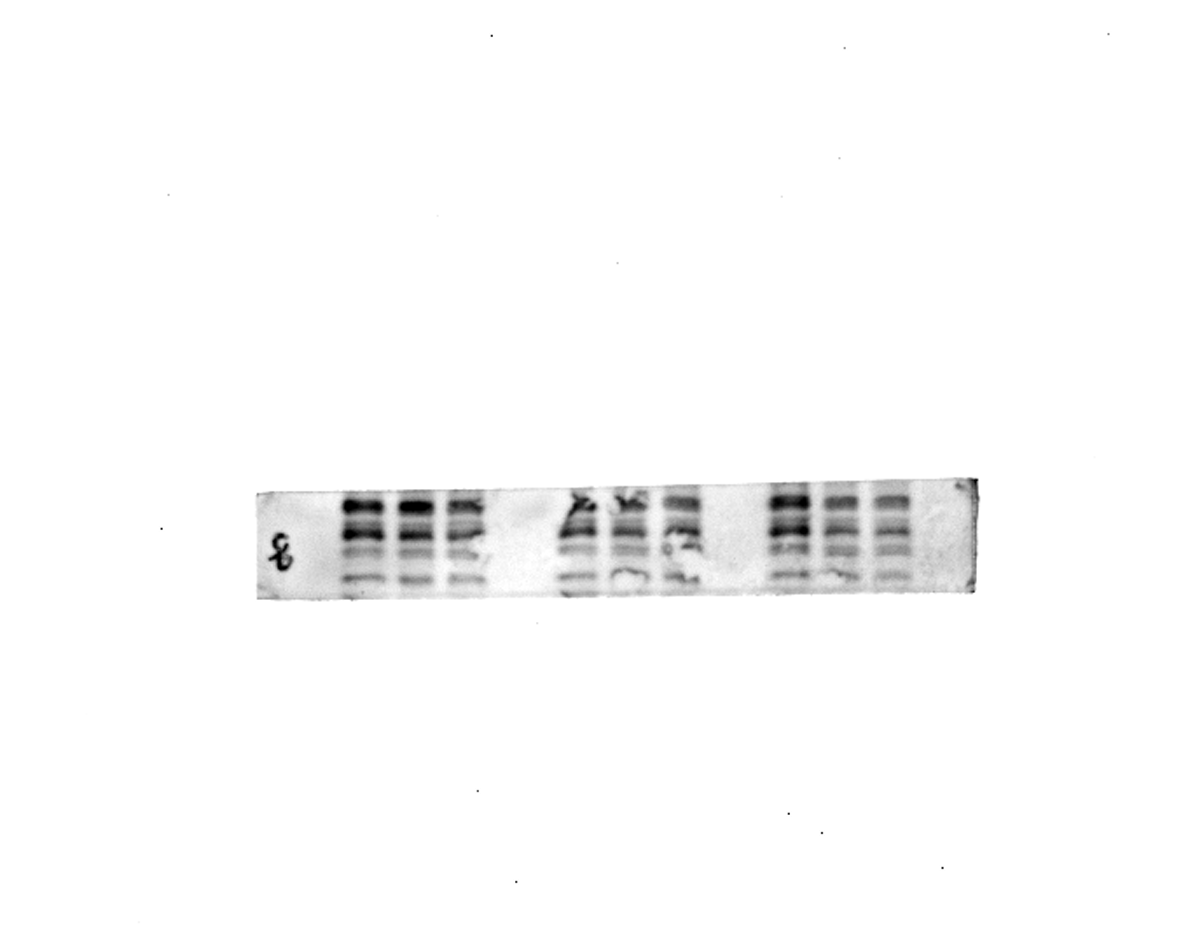

Supplement: Supplementary file 1 [file cancers-15-00027-s001.zip › cancers-2083385-File S2/Figure 8B-8C/C/Snail-bel7404-shSTX6.tif]

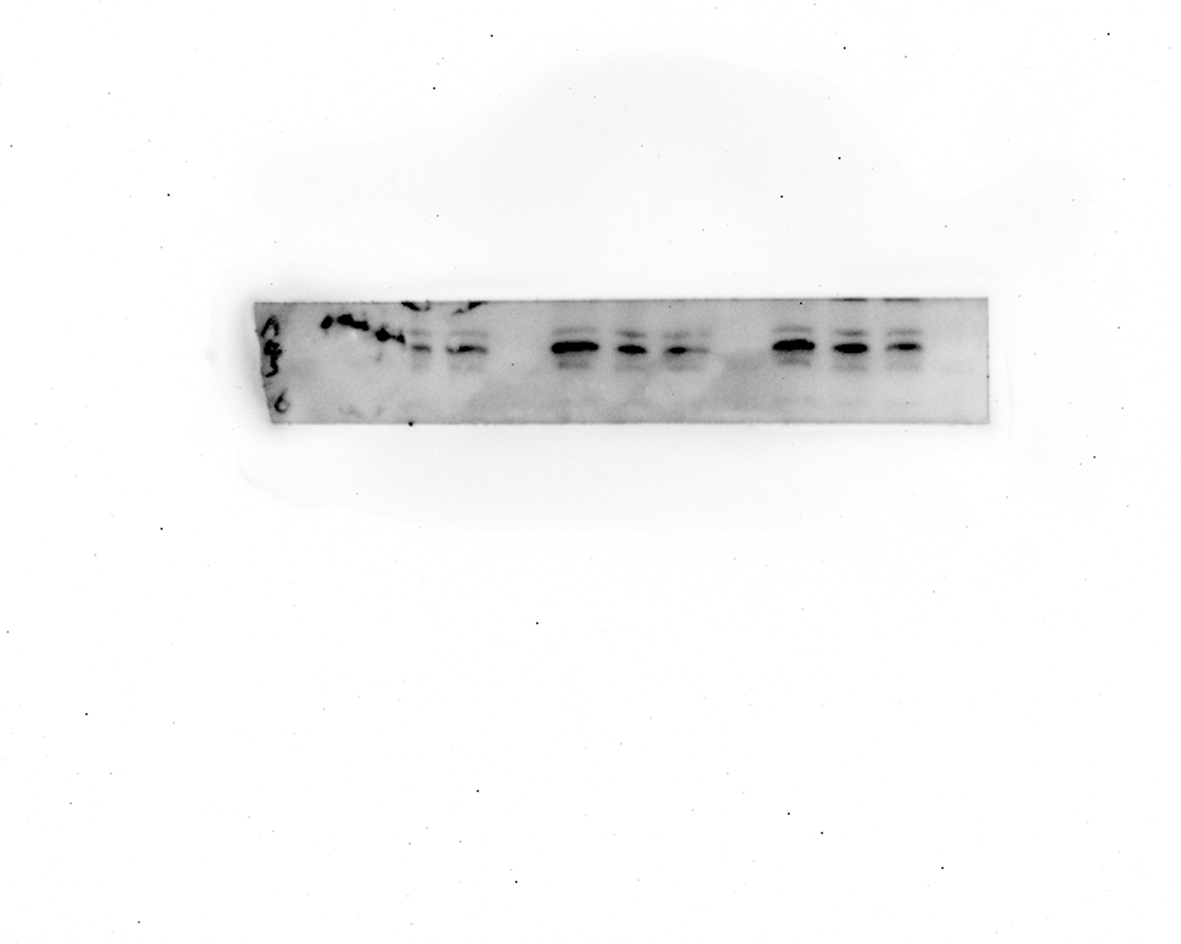

Supplement: Supplementary file 1 [file cancers-15-00027-s001.zip › cancers-2083385-File S2/Figure 8B-8C/C/snail-shSTX6-HCT-116.tif]

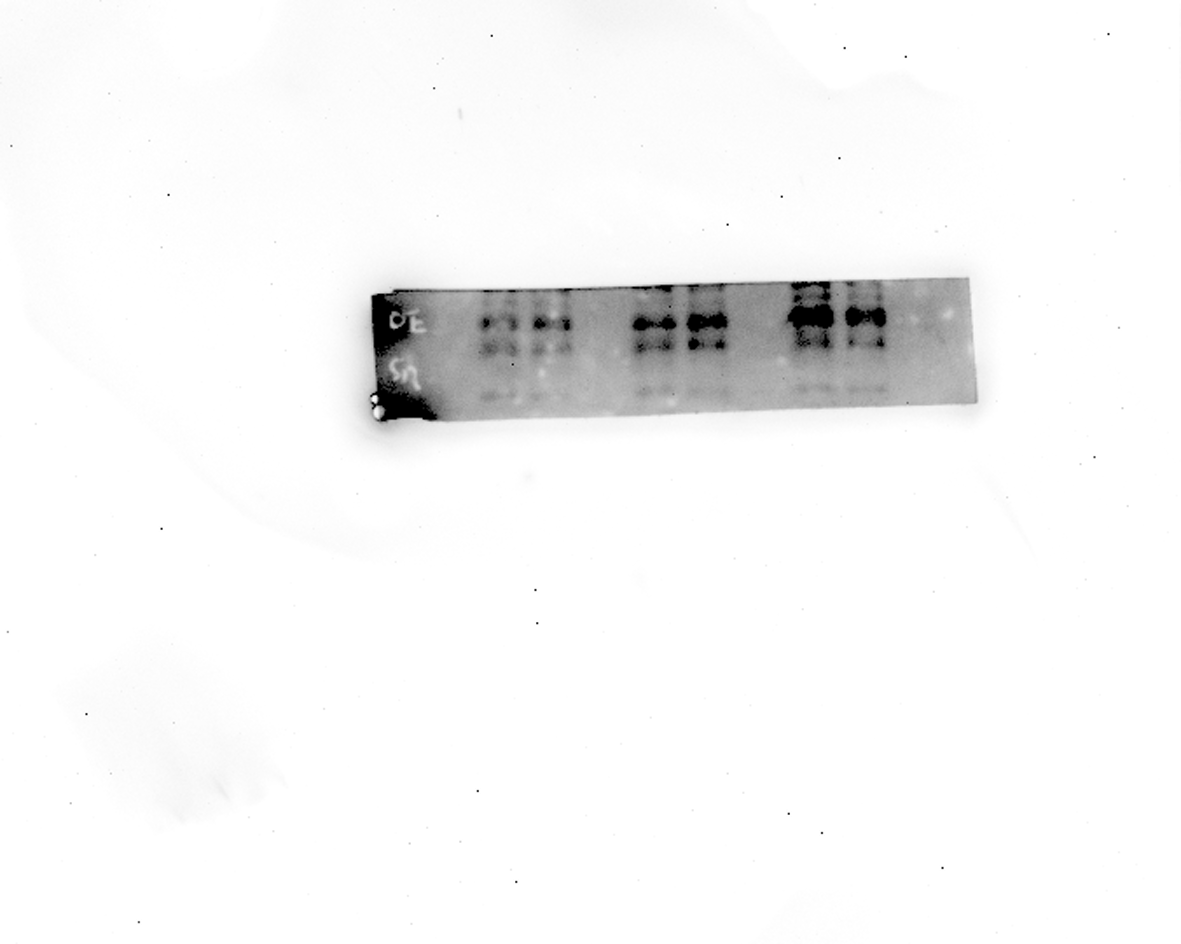

Supplement: Supplementary file 1 [file cancers-15-00027-s001.zip › cancers-2083385-File S2/Figure 8B-8C/C/Snail-SW480-OE.tif]

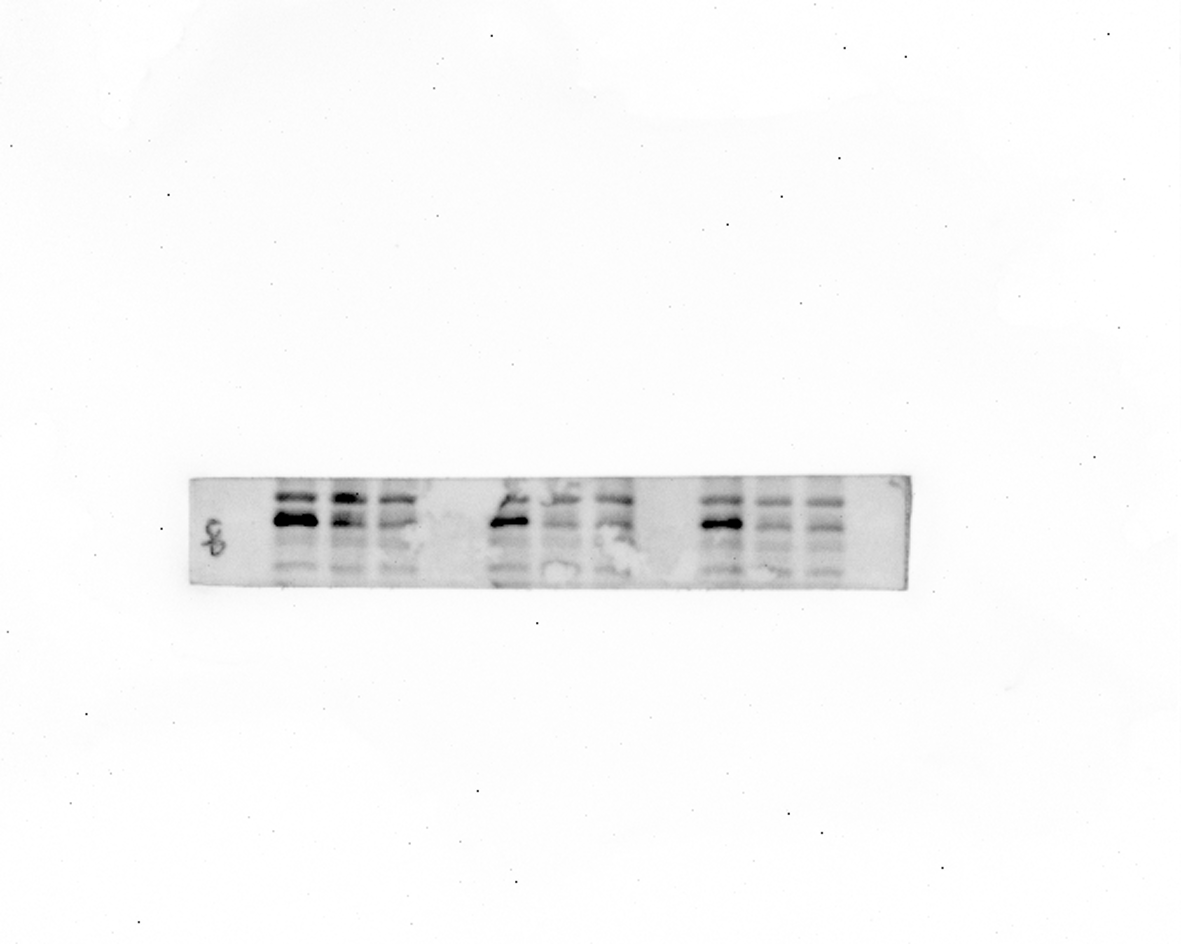

Supplement: Supplementary file 1 [file cancers-15-00027-s001.zip › cancers-2083385-File S2/Figure 8B-8C/C/stx6-BEL7404-shSTX6.tif]

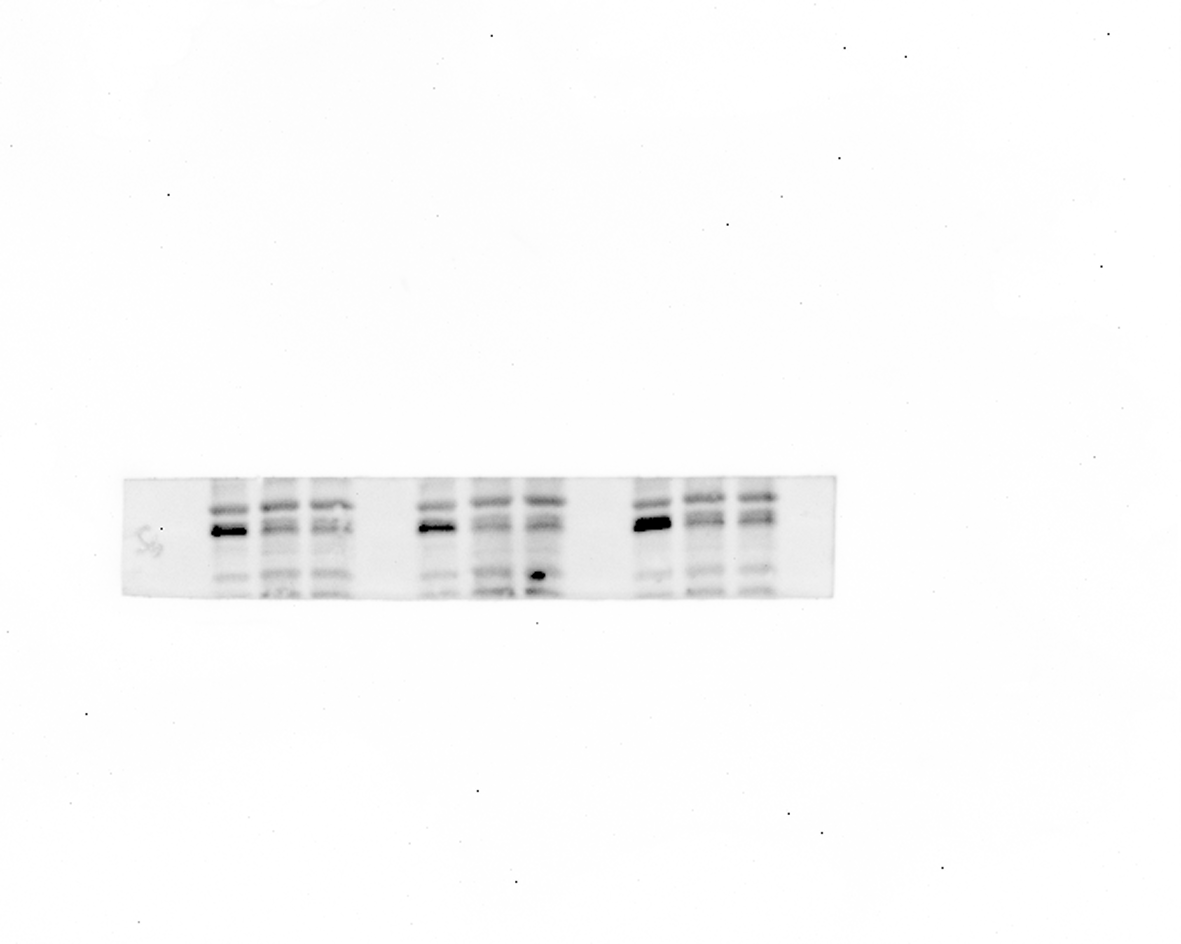

Supplement: Supplementary file 1 [file cancers-15-00027-s001.zip › cancers-2083385-File S2/Figure 8B-8C/C/stx6-hct116-shSTX6.tif]

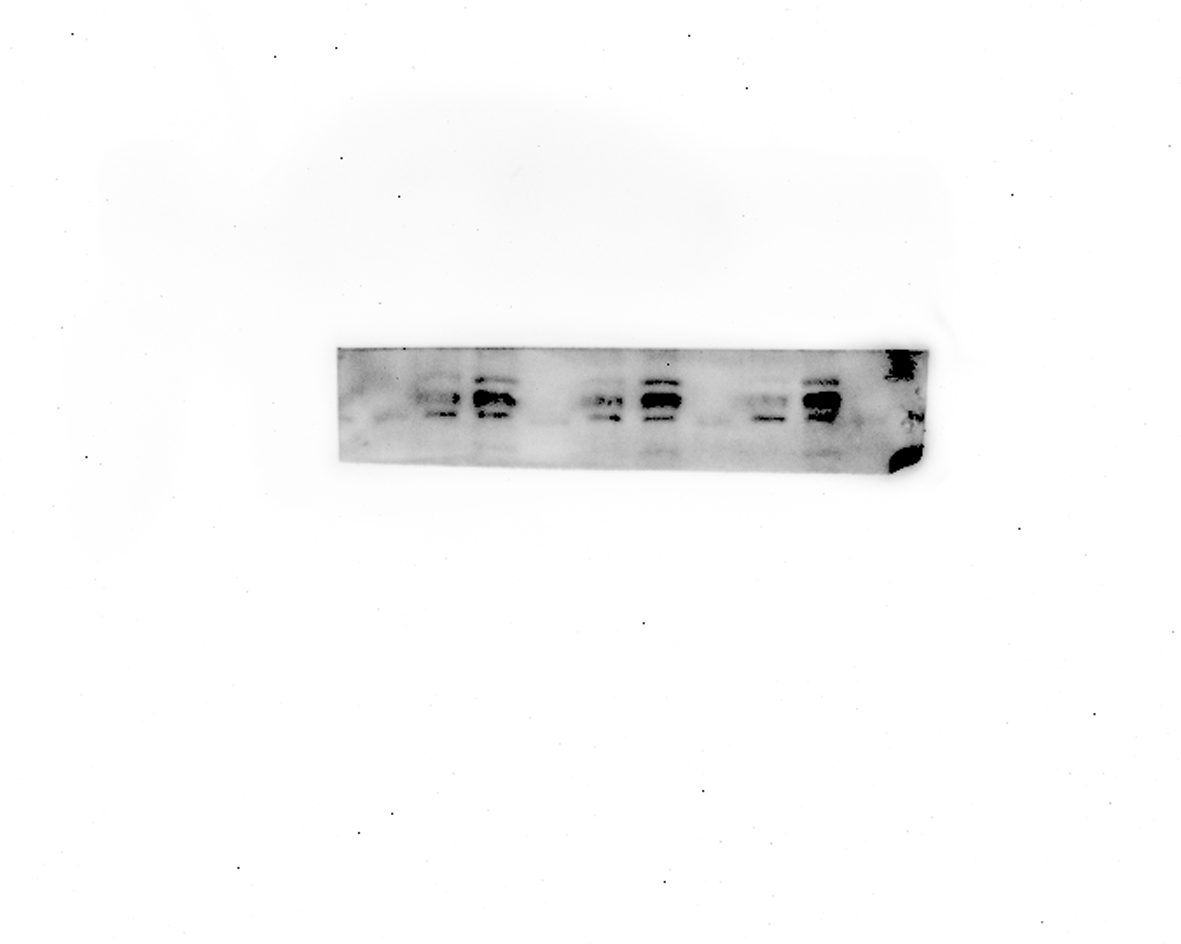

Supplement: Supplementary file 1 [file cancers-15-00027-s001.zip › cancers-2083385-File S2/Figure 8B-8C/C/STX6-SW480-OE.tif]

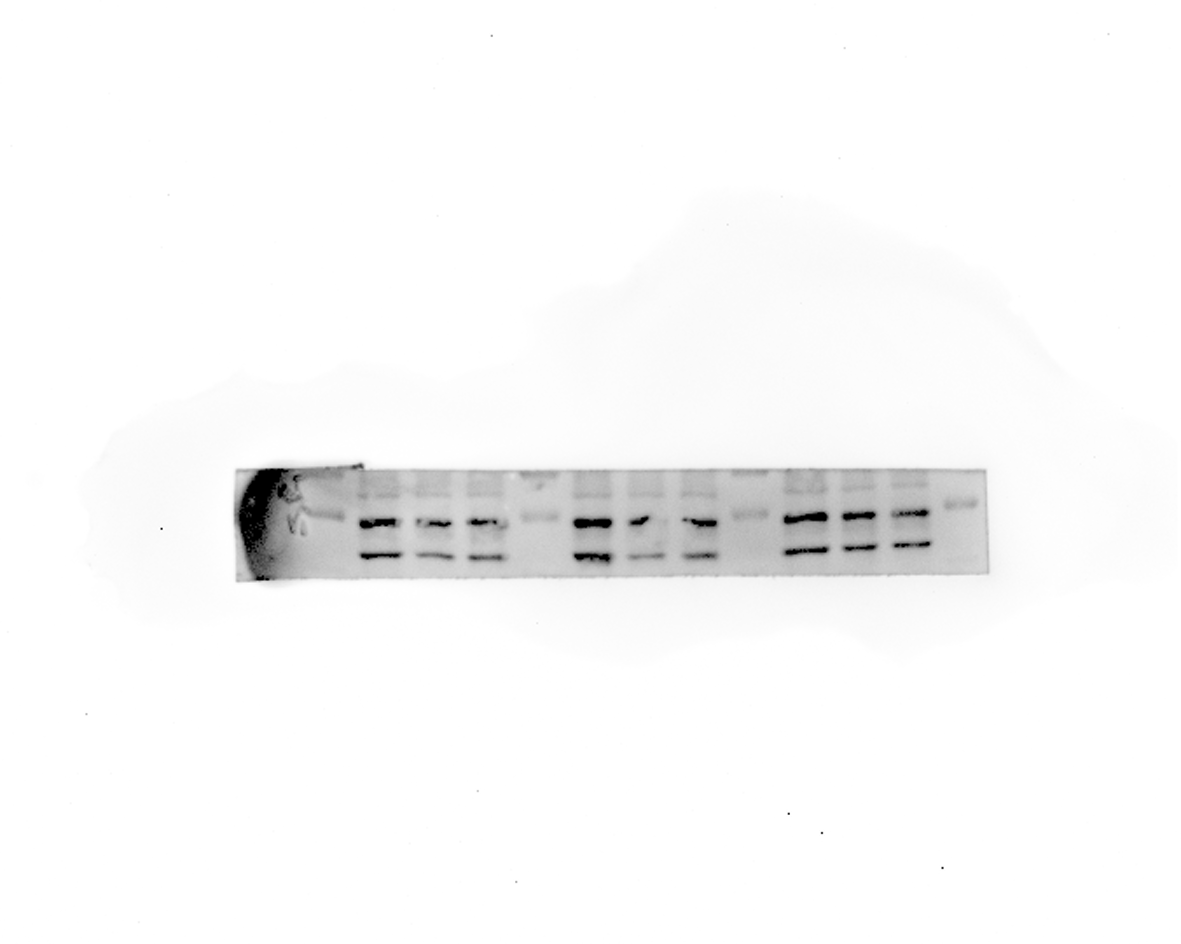

Supplement: Supplementary file 1 [file cancers-15-00027-s001.zip › cancers-2083385-File S2/Figure 8B-8C/C/Vintenmin-BEL7404-shSTX6.tif]

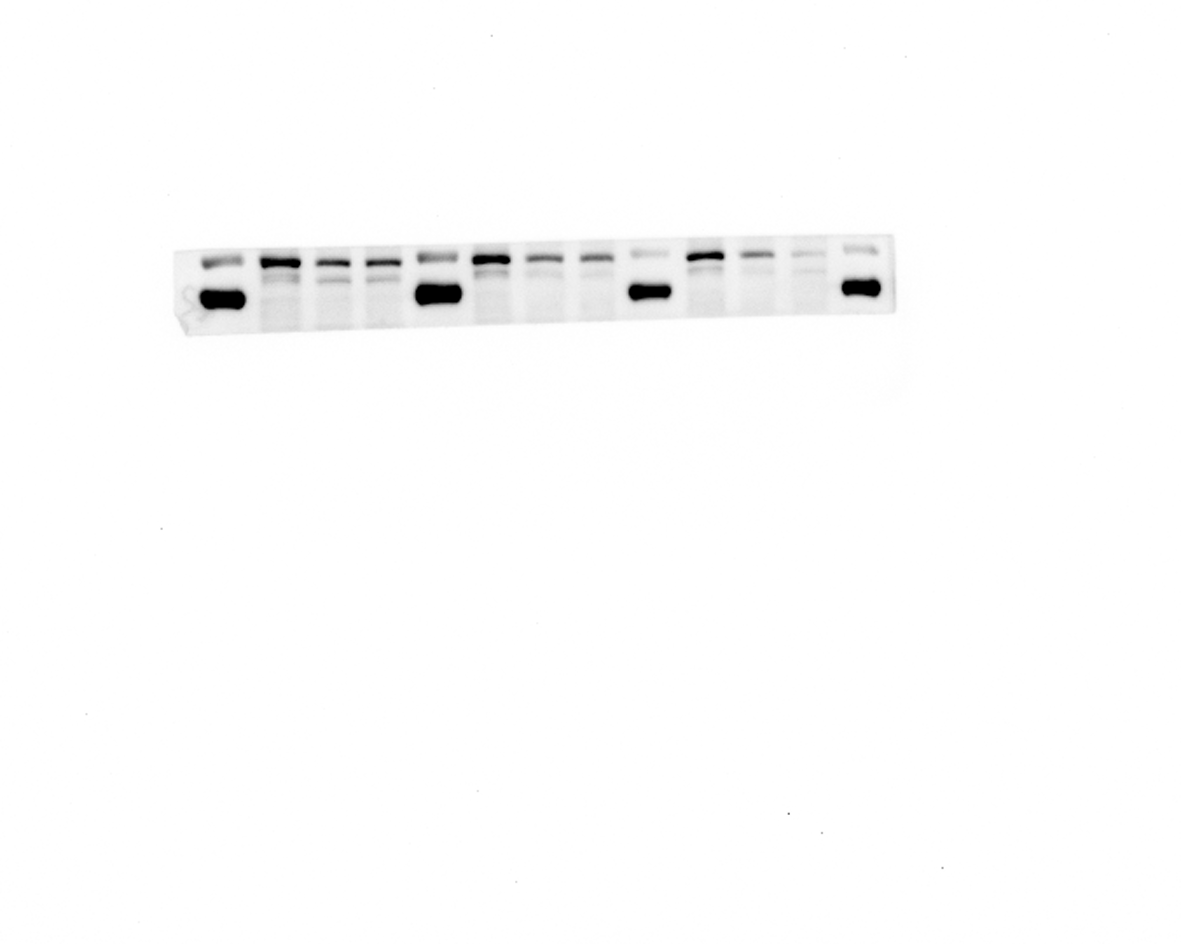

Supplement: Supplementary file 1 [file cancers-15-00027-s001.zip › cancers-2083385-File S2/Figure 8B-8C/C/Vintenmin-HCT116-shSTX6.tif]

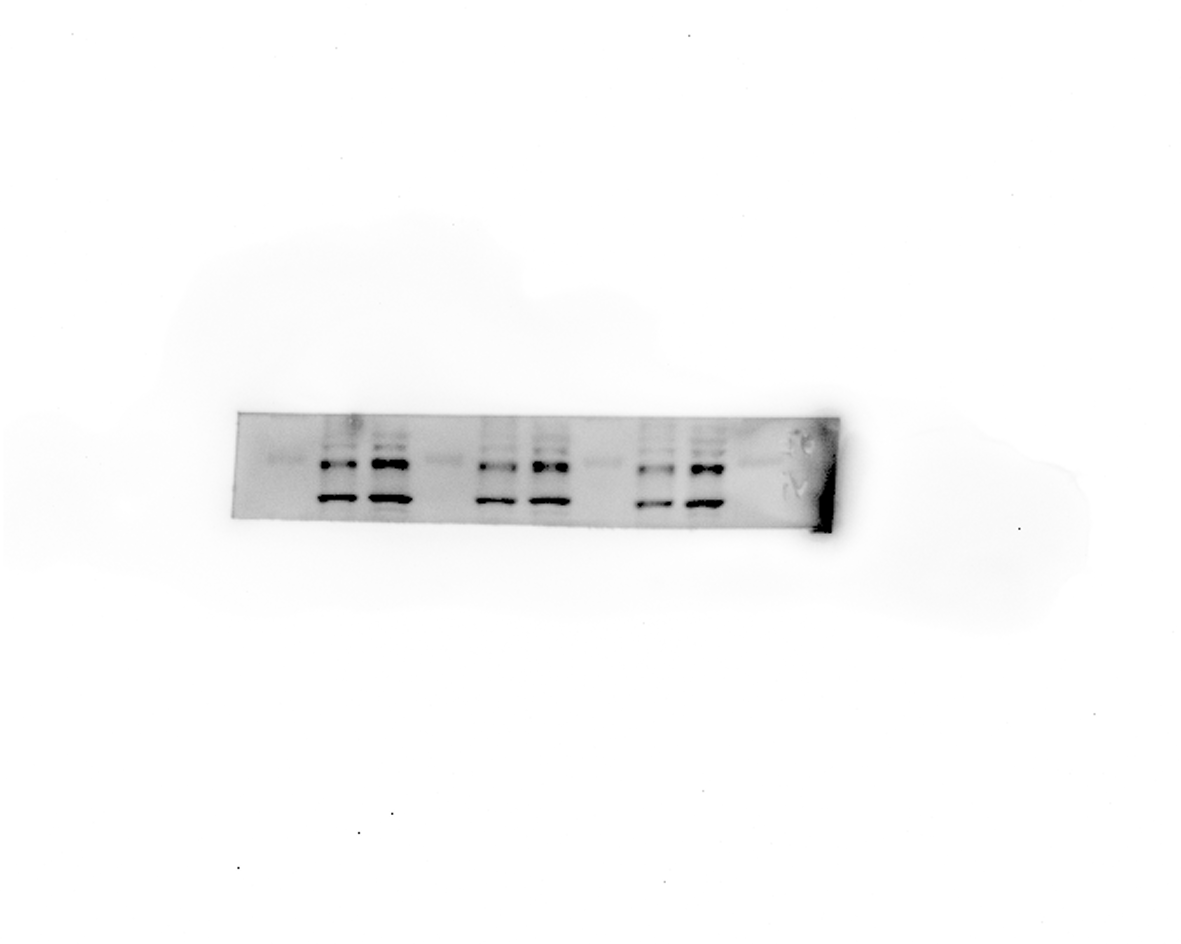

Supplement: Supplementary file 1 [file cancers-15-00027-s001.zip › cancers-2083385-File S2/Figure 8B-8C/C/Vintenmin-SW480-OE.tif]

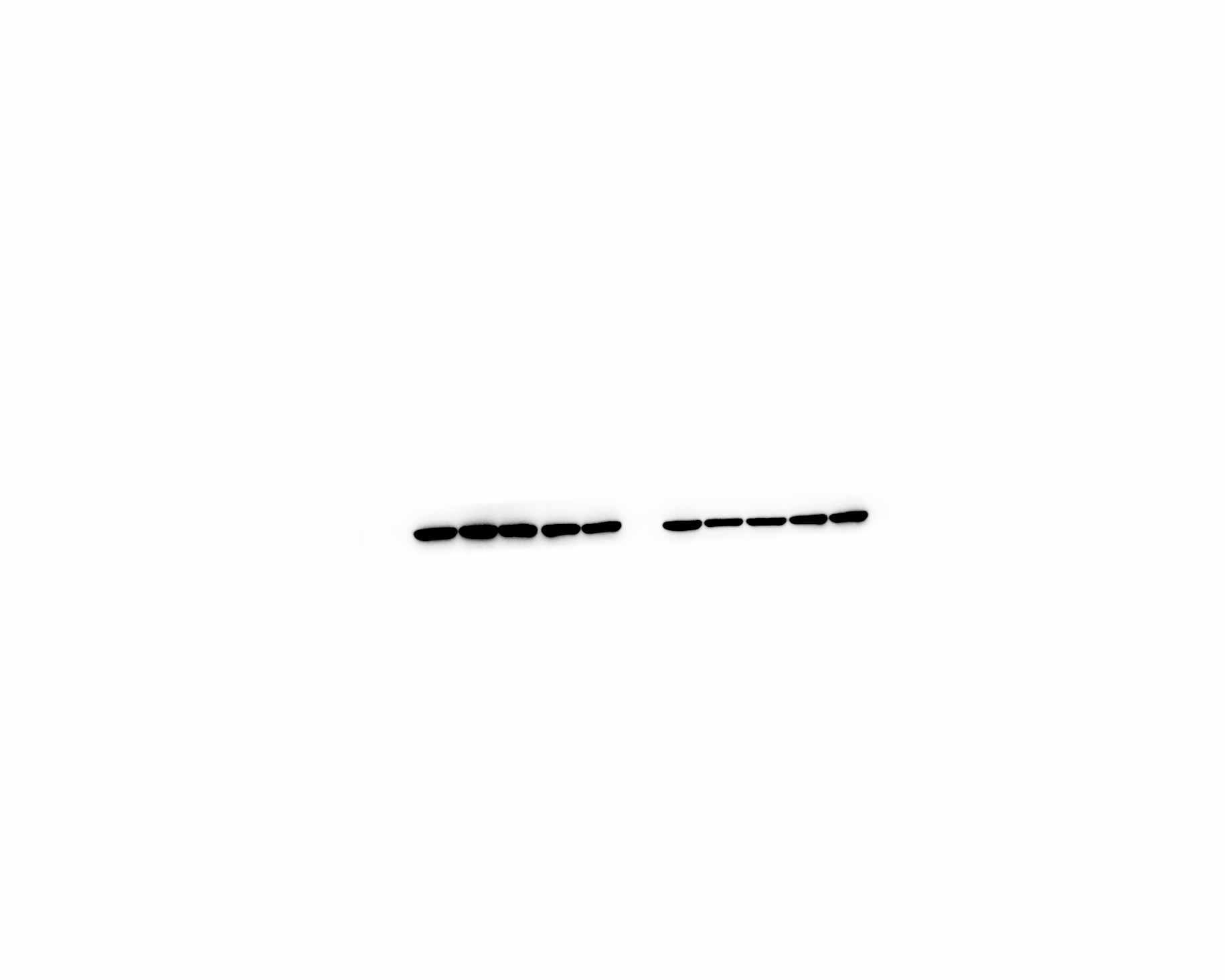

Supplement: Supplementary file 1 [file cancers-15-00027-s001.zip › cancers-2083385-File S2/Figure7C-7D/ACTIN-CRC cell line.tif]

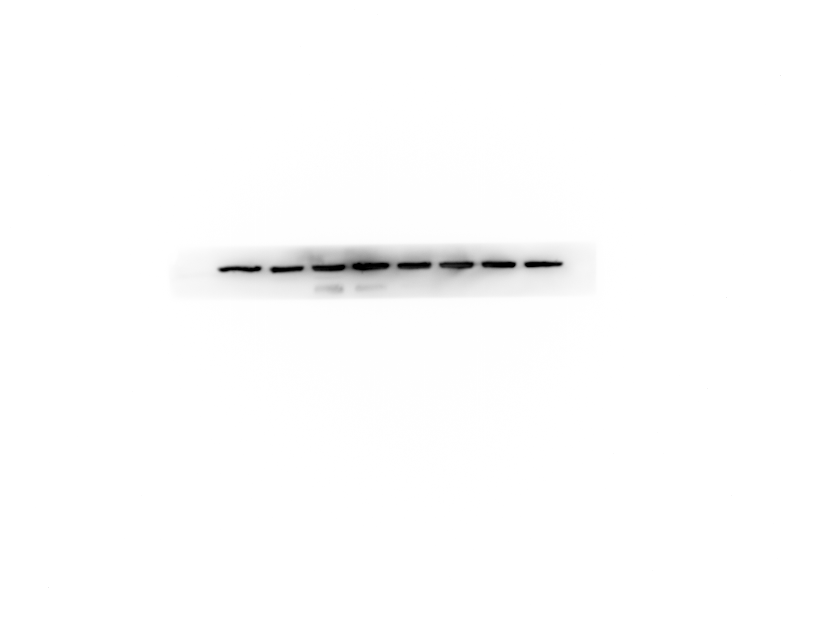

Supplement: Supplementary file 1 [file cancers-15-00027-s001.zip › cancers-2083385-File S2/Figure7C-7D/Actin-HCC-cell lines.png]

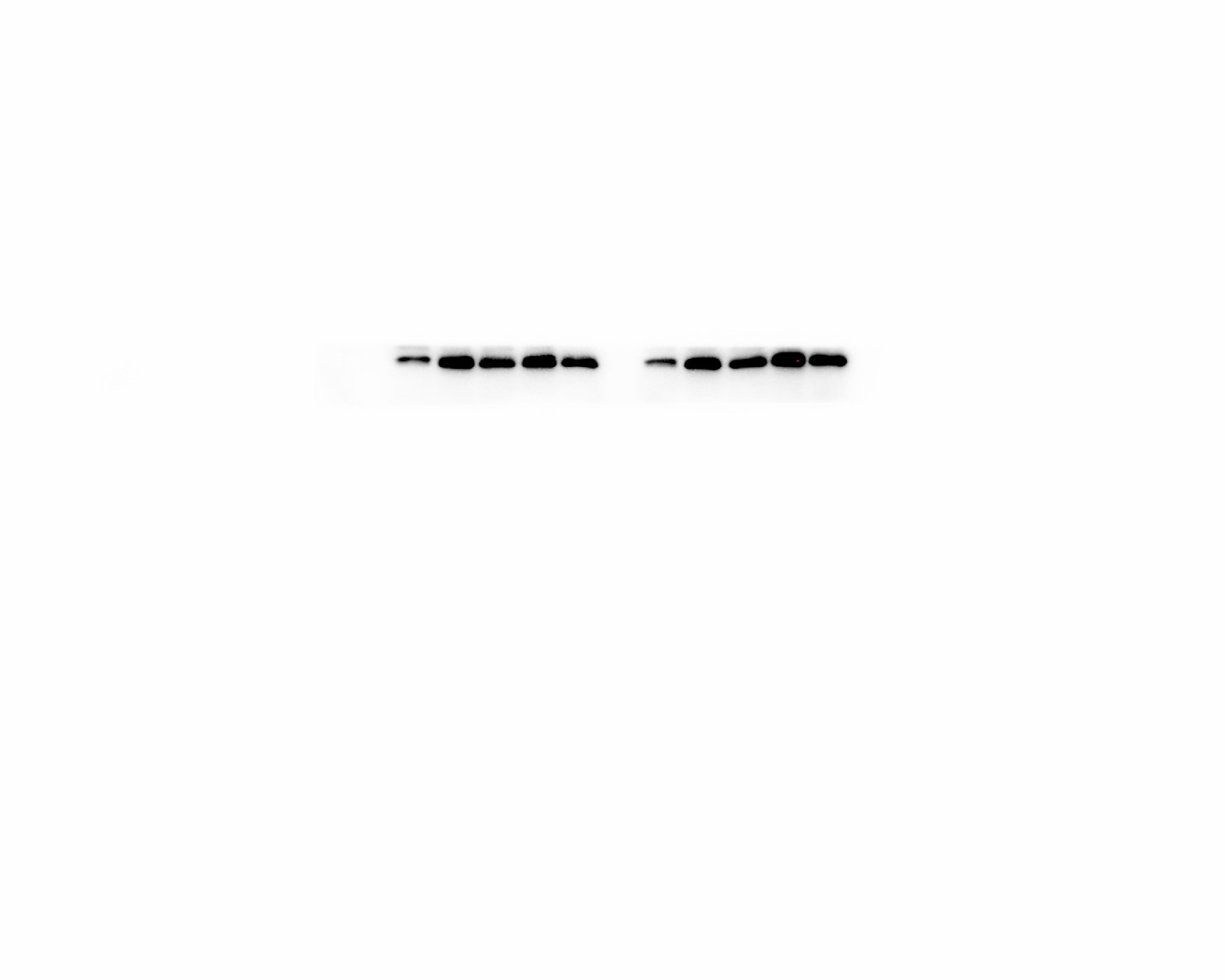

Supplement: Supplementary file 1 [file cancers-15-00027-s001.zip › cancers-2083385-File S2/Figure7C-7D/STX6-CRC cell line.jpg]

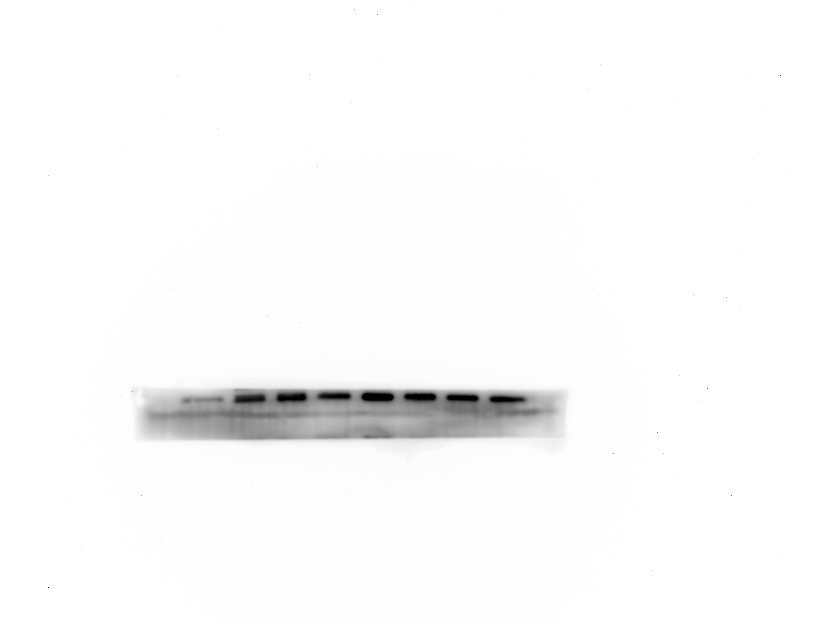

Supplement: Supplementary file 1 [file cancers-15-00027-s001.zip › cancers-2083385-File S2/Figure7C-7D/STX6-HCC-cell lines.png]
